# Microbiota restoration reduces antibiotic-resistant bacteria gut colonization in patients with recurrent *Clostridioides difficile* infection from the open-label PUNCH CD study

**DOI:** 10.1186/s13073-021-00843-9

**Published:** 2021-02-16

**Authors:** Amy Langdon, Drew J. Schwartz, Christopher Bulow, Xiaoqing Sun, Tiffany Hink, Kimberly A. Reske, Courtney Jones, Carey-Ann D. Burnham, Erik R. Dubberke, Gautam Dantas, for the CDC Prevention Epicenter Program

**Affiliations:** 1 The Edison Family Center for Genome Sciences & Systems Biology, Washington University School of Medicine in St. Louis, St. Louis, MO USA; 2 Clinical Research Training Center, Washington University School of Medicine in St. Louis, St. Louis, MO USA; 3 Department of Pediatrics, Washington University School of Medicine in St. Louis, St. Louis, MO USA; 4 Department of Pathology and Immunology, Division of Laboratory and Genomic Medicine, Washington University School of Medicine in St. Louis, St. Louis, MO 63110 USA; 5 Department of Medicine, Washington University School of Medicine in St. Louis, St. Louis, MO 63110 USA; 6 Rebiotix, Inc., Minneapolis, MN USA; 7 Department of Molecular Microbiology, Washington University School of Medicine in St. Louis, St. Louis, MO 63110 USA; 8 Department of Biomedical Engineering, Washington University in St Louis, St. Louis, MO USA

**Keywords:** Fecal microbiota transplantation, Multidrug resistance, Antibiotic resistance, Metagenomics, Microbiome, *Clostridioides difficile*

## Abstract

**Background:**

Once antibiotic-resistant bacteria become established within the gut microbiota, they can cause infections in the host and be transmitted to other people and the environment. Currently, there are no effective modalities for decreasing or preventing colonization by antibiotic-resistant bacteria. Intestinal microbiota restoration can prevent *Clostridioides difficile* infection (CDI) recurrences. Another potential application of microbiota restoration is suppression of non-*C. difficile* multidrug-resistant bacteria and overall decrease in the abundance of antibiotic resistance genes (the resistome) within the gut microbiota. This study characterizes the effects of RBX2660, a microbiota-based investigational therapeutic, on the composition and abundance of the gut microbiota and resistome, as well as multidrug-resistant organism carriage, after delivery to patients suffering from recurrent CDI.

**Methods:**

An open-label, multi-center clinical trial in 11 centers in the USA for the safety and efficacy of RBX2660 on recurrent CDI was conducted. Fecal specimens from 29 of these subjects with recurrent CDI who received either one (*N* = 16) or two doses of RBX2660 (*N* = 13) were analyzed secondarily. Stool samples were collected prior to and at intervals up to 6 months post-therapy and analyzed in three ways: (1) 16S rRNA gene sequencing for microbiota taxonomic composition, (2) whole metagenome shotgun sequencing for functional pathways and antibiotic resistome content, and (3) selective and differential bacterial culturing followed by isolate genome sequencing to longitudinally track multidrug-resistant organisms.

**Results:**

Successful prevention of CDI recurrence with RBX2660 correlated with taxonomic convergence of patient microbiota to the donor microbiota as measured by weighted UniFrac distance. RBX2660 dramatically reduced the abundance of antibiotic-resistant Enterobacteriaceae in the 2 months after administration. Fecal antibiotic resistance gene carriage decreased in direct relationship to the degree to which donor microbiota engrafted.

**Conclusions:**

Microbiota-based therapeutics reduce resistance gene abundance and resistant organisms in the recipient gut microbiome. This approach could potentially reduce the risk of infections caused by resistant organisms within the patient and the transfer of resistance genes or pathogens to others.

**Trial registration:**

ClinicalTrials.gov, NCT01925417; registered on August 19, 2013.

**Supplementary Information:**

The online version contains supplementary material available at 10.1186/s13073-021-00843-9.

## Background

Antibiotic-resistant (AR) infections account for billions of dollars in healthcare costs and tens of thousands of deaths every year in the USA alone [1]. Infections caused by antibiotic-resistant organisms (AROs) are even more devastating because of dwindling therapeutic options. Increasing global usage of antibiotics raises the abundance and prevalence of antibiotic resistance genes (ARGs) and AROs both within an individual and the environment [2–5]. Even when appropriately delivered, antibiotics disrupt the commensal gut microbiota, select for antibiotic resistance, and decrease colonization resistance to AROs and opportunistic pathogens [6–8]. Therefore, development and implementation of antibiotic-sparing alternatives is imperative to limit the sequelae of increased AR worldwide.

Antibiotic treatment increases the risk of *Clostridioides difficile* infection (CDI) by decreasing colonization resistance mediated by commensal organisms [9, 10]. Currently, CDI is primarily treated with orally bioavailable antibiotics such as vancomycin or metronidazole, which further contributes to microbiome disruption, AR infections, and risk for recurrent CDI [11–13]. Furthermore, antibiotic treatment with metronidazole and vancomycin increases the carriage of AROs such as vancomycin-resistant *Enterococci* (VRE) [14]. Increased gastrointestinal carriage of VRE in the context of *C. difficile* colitis can predispose patients to VRE bacteremia with 2.5-fold increased mortality relative to vancomycin-sensitive *Enterococci* [15, 16]. Thus, development of antibiotic-sparing treatments to restore gut microbiota composition, enhance colonization resistance, and limit increasing antibiotic resistance is warranted.

Fecal microbiota transplantation (FMT) is a technique whereby donor stool from healthy individuals is delivered into the gastrointestinal tract of a recipient patient. FMT is rapidly gaining recognition as a mostly safe and highly effective treatment for preventing recurrent CDI [13, 17, 18], and analogous investigational microbiota-based therapeutics are under evaluation in controlled clinical trials [19, 20]. Additionally, these approaches have the potential to restore other aspects of a disrupted gut microbiome [21]. Indeed, previous studies have demonstrated taxonomic changes to the gut microbiota via 16S rRNA gene sequencing after FMT for recurrent CDI commensurate with an increase in gut microbial diversity, a marker of microbiota health [12, 13]. While some patients respond well to a single FMT, some require repeat FMTs to prevent CDI recurrence, and it is accordingly important to be able to predict engraftment success [22]. It was recently shown that probability of bacterial species engraftment after FMT was related to the taxonomic abundance of each species in the donor and in the recipient [22]. Some studies also suggest that there may be a reduction in carriage of ARGs and selected AROs such as VRE after FMT [23, 24]. It is therefore theoretically possible to utilize FMT or similar investigational treatments with a high abundance of non-resistant species to displace AROs from the recipient’s microbiome. Accordingly, we sought to investigate the abundance of AROs and ARGs in patients treated with RBX2660—a microbiota-based investigational therapeutic for alleviation of recurrent CDI.

RBX2660, a liquid suspension of donor microbiota screened for bacterial, viral, and parasitic pathogens, including methicillin-resistant *Staphylococcus aureus* (MRSA), vancomycin-resistant *Enterococci* (VRE), and extended-spectrum beta lactamase (ESBL) -expressing *Enterobacteriaceae*, has recently been deployed to treat recurrent CDI [25, 26]. Here we examine the effects of this treatment on the recipient’s microbiome, ARG prevalence within the gut, and the fates of patient-derived ARO isolates over the course of a 12-week phase II clinical trial, and up to 180 days post-therapy. We found that patients who adopt a more donor-like microbiota composition, determined by weighted UniFrac distance 7 days after RBX2660, were more likely to be CDI recurrence-free during the 180-day observation period. We tracked ARO abundance in the recipient’s stool after initial therapy via longitudinal strain tracking of amplicon sequence variants (ASVs) based on the 16S rRNA gene sequences of cultured isolates. We utilized whole metagenome shotgun sequencing and ARG prediction using ShortBRED to quantify ARGs in the recipient, which we find is correlated with weighted UniFrac distance from the donor. Taken together, these data show that in addition to CDI treatment, RBX2660, and potentially FMT in general, can be used to reduce overall ARG abundance and ARO carriage in a recipient’s microbiome.

## Methods

### Trial design

Fecal samples in this study were derived from a phase 2 prospective open-label cohort study administering the microbiota-based restoration therapeutic RBX2660 to patients with recurrent CDI (NCT01925417). Safety and efficacy analysis of this trial has been published [25], and the study protocol is detailed there and reproduced here (Additional file 1: Fig. S1). The first patient was enrolled on August 15, 2013, and the last was enrolled on December 16, 2013. Forty patients were recruited at 11 study sites within the USA. For inclusion, patients 18 years or older had at least two rounds of standard-of-care oral antibiotic therapy with at least two recurrences or hospitalizations for CDI. They also had to take or start oral antibiotics for CDI symptoms including at least 7 days of oral vancomycin. Exclusion criteria included medical diagnoses and procedures that could rationally impact the gut microbiome including uncontrolled diarrhea after CDI treatment, concurrent antibiotic therapy for an illness other than CDI, or history of inflammatory bowel disease, irritable bowel syndrome, chronic diarrhea, or celiac disease [25]. Patients with compromised immune systems including steroid use, neutropenia, chemotherapy, or a life expectancy less than 12 months were also excluded. The primary outcome was incidence of serious adverse events through 56 days after the last treatment. Secondary outcomes included incidence of serious adverse events 6 months after the last treatment, absence of CDI 56 days after the last dose, quality of life score, and hospitalization data after RBX2660. Prior to administration of the study drug, all patients were given at least 7 days of oral vancomycin (125 mg four times per day) followed by RBX2660 via enema from one of 21 samples from four healthy donors. Of the 34 patients that passed screening, 29 succeeded in submitting longitudinal fecal samples suitable for microbiome analysis. If a patient had a recurrence of CDI symptoms, they were offered a second dose of RBX2660. The study population was 97% white and 67.6% female and had a mean age of 68 years [25].

### Study drug

The microbiota-based restoration therapeutic RBX2660 is a 50-g/150-mL suspension of donor stool containing at least 10^7^ CFU live microbes in polyethylene glycol 3350/0.9% sodium chloride USP solution. The donor stools were screened extensively for MRSA and VRE as well as viral, bacterial, and parasitic enteric pathogens as previously described [25]. Aliquots of all 21 RBX2660 products from 4 donors were retained and utilized for this study.

### Sample collection

**Fig. 1:**
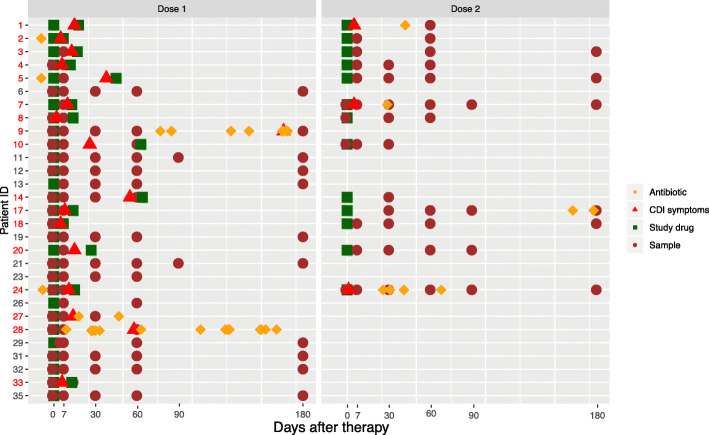
Sampling schematic. Patients were given RBX2660 (green square) after vancomycin oral therapy (left panel). Stools (labeled as maroon circles) provided were sequenced and used for subsequent analyses. If a patient had CDI recurrence (red triangle), they were offered a second dose of RBX2660 (green square) with subsequent stools provided after the second study drug (right panel). Any antibiotic treatment during the trial is labeled as yellow diamonds. Patient IDs colored red failed first treatment and received antibiotics or second dose and constitute the RI group (*n* = 17). Patients who had no recurrence of symptoms or received antibiotics were considered successes (SI group, *n* = 12). All subsequent figures utilize data after the first dose. Data after second RBX2660 is used only for Figs. 5a–e and 6d. Three stool samples that failed sequencing were excluded from this figure and downstream analyses

Stool samples were collected at day 0 (after finishing vancomycin treatment and before RBX2660 administration), and at days 7, 30, 60, 90, and 180 post-treatment, though many patients did not provide all samples (Fig. 1). Samples were collected at home by patients and immediately shipped on ice (4 °C) in dedicated sterile, airtight containers via FedEx. The stool samples were divided into 500-mg aliquots that were placed at − 80 °C immediately upon receipt by Rebiotix, Inc. Samples were shipped from Rebiotix, Inc. on dry ice (− 20 °C) to Washington University in St. Louis, Missouri.

### DNA extraction and sequencing

Fecal DNA was extracted from 0.25 g of stool via phenol-chloroform extraction as follows. Stool was combined with 250 μL of 0.1 mm zirconium beads, 500 μL of 200 mM NaCl/200 mM Tris/20 mM EDTA solution, 210 μL of 20% SDS buffer, and 500 μL 24:25:1 phenol to chloroform to IAA (pH 7.9) while on ice. This mixture was homogenized via bead beating for 4 min and then centrifuged at 4 °C for 3 min at 6800rcf. The aqueous supernatant was transferred to pre-spun lock-phase PLG columns (5Prime, #2302820), an equivalent volume of phenol to chloroform to IAA was added, and the tube was inverted and then centrifuged at max speed (20,800 rcf) for 5 min. The aqueous phase was transferred to a clean tube with 600 μL of cold isopropanol and 60 μL of 3 M NaOAc (pH 5.5), mixed, and incubated at − 20 °C overnight. The resultant precipitate was pelleted by centrifugation at 20,800 rcf at 4 °C for 20 min. The supernatant was decanted, and the pellet was washed by adding 500 μL of 100% EtOH at 25 °C, centrifuging at 20,800 rcf at 4 °C for 3 min. The ethanol was pipetted off, and the pellet was air-dried for 15 min in dark, sterile conditions. Finally, the pellet was resuspended in 50 μL of TE buffer (Ambien #9861) while incubating at 30 °C for 5 to 15 min. The resulting DNA was processed with QIAQuick PCR purification column (QIAGEN #28106) with 4 μL of 100 mg/mL RNase added to 300 μL of Buffer PB at step 1 and incubated with the resuspended DNA for 2 min at room temperature.

The 16S rRNA gene was amplified from fecal DNA as follows: 1.5 ng of fecal DNA was used as template for PCR reactions using 5PRIME HotMasterMix (Quantabio #22000401) with universal 16S rRNA gene primers 515F (5′-GTGCCAGCMGCCGCGGTAA) and 806R (5′-GGACTACHVHHHTWTCTAAT). An 8-bp barcode unique to each sample was designated, and each reaction was run in triplicate with a negative (no template) control. The amplicons were run on a 1% TAE agarose gel with SYBRsafe DNA stain and gel-purified with Qiagen Gel Purification Kit (#28115). Eluted amplicons were quantified with PicoGreen dsDNA (ThermoFisher #P7581), pooled, and purified with Agencourt AMPure XP bead purification protocol per the manufacturer’s instructions (Beckman Coulter #A63881). The pool was loaded at 8 pM concentration with 25% PhiX and sequenced on the Illumina MiSeq platform with 2 × 150 bp paired end reads.

For whole metagenome shotgun sequencing, 130 μL containing at least 500 ng of genomic DNA was sonicated (Covaris E220 model) into 500–600-bp fragments at 4 °C for 75 s, at intensity 4, duty cycle 10%, and 200 cycles per burst. Fragmented DNA was concentrated into 63 μL volume using the QIAQuick PCR Purification kit (Qiagen). End repair was performed using 0.5 μL of three enzymes: T4 ligase (NEB #M0203S), Taq polymerase (NEB #M0267S), and T4 PNK (NEB #M0201S), with 1 μL of 1 mM dNTPs and 2.5 μL of T4 buffer with 10 mM ATP (NEB #B0202S). The end-repaired genomic fragments were barcoded by incubating the DNA mixture with 0.8 μL of T4 DNA ligase and a unique sequencing barcode at 25 °C for 10 min. Samples were then pooled by column of the 96-well plate, purified by QiaQuick PCR purification kit, and eluted in 15 μL of EB. Gel purification was similar to 16S rRNA gene sequencing but for all fragments from 400 to 900 bp in length, and final elution volume was 12 μL. Finally, 2 μL of each of the shotgun fragment pools was amplified using 1 μL of 10 μM Illumina nonspecific primers using 2X Phusion HF Master Mix and water up to 25 μL total reaction volume with the following cycling conditions: 17× for 30 s each of 98 °C, 65 °C, and 72 °C with a 5 min 72 °C final extension and hold at 4 °C. The product was then quantified by QuBit and pooled at equal concentrations. Purified libraries were then prepared for sequencing on the Illumina HiSeq platform with paired end reads of 2 × 150 bp. Metagenomic shotgun sequencing samples were re-sequenced if the associated barcodes appeared in fewer than 1M reads [27]. Samples that failed sequencing were excluded from analysis and removed from Fig. 1.

### Isolation and genomic analysis of AROs

In order to determine the fates of specific AR bacterial strains, each fecal sample was plated on selective and differential media as described below. Frozen samples were thawed once before DNA extraction in order to aliquot 1 mL for culture. Stool was incubated for 2 h in Tryptic Soy Broth at 35 °C, and two drops of the stool/broth mixture were streaked onto each of the following plates: Sheep’s Blood Agar (SBA) (BD 22161), VRE ChromeID (Biomerieux 43851), MacConkey with Cefotaxime (Hardy G121), CHROME ESBL (Hardy G321), Hardy Cetrimide Agar (Hardy G18), and MRSA Spectra Agar (Remel 01822). An incubator with 5% CO_2_ atmosphere was used for SBA, while the rest were incubated at standard atmospheric compositions and grown at 37 °C. For each selective plate, 4 colonies were chosen for isolation. These colonies were subcultured to an SBA plate and labeled A–D. Each colony was determined to the genus or species level by VITEK MALDI-TOF MS (KB v3.2.0), then subjected to antimicrobial susceptibility testing where it was categorized according to clearance zone diameter cutoffs from CLSI 2016 guidelines [28]. All isolates were stored in − 80 °C in Tryptic Soy Broth with 10% glycerol.

The Qiagen Bacteremia kit was used to extract genomic DNA from 0.25-g bacterial mass from pure culture using the manufacturers’ instructions. Shotgun sequencing was performed as above with each isolate at 100× coverage of the estimated genome size. Genomes were assembled with spades v3.10 (kmer sizes 21, 33, 55, and 77 on careful mode) and quality controlled with QUAST v4.5 [29]. ARGs from isolate genomes were annotated using Resfinder 4.0 [30]. Core genes were extracted with Prokka v1.12 [31] and then aligned and compared using Roary v3.12.0 [32]. Phylogenetic trees were generated from core binary genes using RaxML v8.2.11 [33] with the GTR Gamma model with name derived from DADA2 ASV (see the next section). A phylogenetic tree was constructed using *Methanobrevibacter* as an outgroup, then trimmed to show closely related outgroups per genus displayed. Visualization was done with the ggtree package in R.

### Isolate tracking in fecal samples using ASVs

The 16S rRNA gene from the isolate shotgun genomes was assembled with PhyloFlash v3.3 with bbmap option [34] and then aligned to the Silva 16S rRNA gene database release 132 (clustering NR99). From the now full-length 16S rRNA gene sequence assembled from each isolate [35], the in silico amplicon from the respective universal 16S rRNA primer was obtained via mothur v1.37.5 [36]. Each of these isolate-derived amplicons was then formatted for inclusion as a pure sample in DADA2 v1.8. The matching ASV was then quantified within patient samples throughout the study.

### Resistance gene prediction and quantification

ShortBRED protein markers were built from the Comprehensive Antibiotic Resistance Database (CARD) 3.0 (February 2019 update) database using shortbred-identify.py with cluster identity 90% and screened against Uniref90 (February 2019 update) [37]. The number of hits for each gene was determined with ShortBRED-quantify, which normalizes reads based on marker length and read depth. A Gaussian linear mixed effects model created using the glmer function of the lmer4 package in R was used to predict ARG totals based on the distance from donor (DFD) metric. The formula for the full model was ARGs ~ DFD + (1 | PatientID), and the fixed effect DFD was restricted to 1 for the null model. In the response variable ARGs, the data was log transformed using glmer option Gaussian (link= “log”) to normalize the right-skewed distribution. For visualization, the *y* axis of ARG totals was expressed as log (ARGS+1) which avoids infinite values. An ANOVA with chisq test comparing the full and null models was run to determine the value of DFD in predicting ARG totals in a metagenome (Chisq = 72.28, d.f.(full) = 1, *p* < 2.2 × 10^−16^).

ARGs were categorized according to the mechanism and then by gene family as available in the CARD 3.0 ontology. Gene families present in at least 10% of samples from either day 0 or day 180 were assessed by the Kruskal-Wallis rank sum test for differences between day 0, day 180, and associated donor samples. The gene families with significant differences by Kruskal-Wallis then underwent pairwise comparison with Wilcoxon rank sum tests with Benjamini-Hochberg correction.

### Taxonomy and microbial functional pathway prediction

**Fig. 2:**
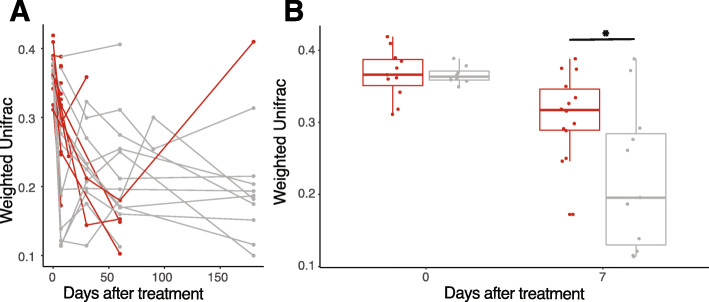
Microbiota composition similarity to the donor at 7 days is predictive of treatment outcome. The donor and recipient microbiota compositions were assessed via 16S rRNA gene sequencing followed by DADA2, and their similarity to the donor product was quantified by weighted UniFrac at each timepoint. **a** Gray lines represent individuals successfully treated with one administration (SI group) while red lines are patients who needed further treatment (RI group, a second product or antibiotics). *N* = 28 patients and 130 samples. **b** Plot demonstrating average distance from the donor at timepoints 0 and 7 days after treatment. *N* = 28 total patients and 44 samples. Box subsumes 75% of the data with a horizontal bar at the median. *p < 0.05, Mann-Whitney *U* test

The annotation of 16S rRNA gene sequences was performed with DADA2 v1.8 with a lower limit read cutoff of 1M reads [38]. Taxonomy was inferred using intrinsic IdTaxa from DADA2 as well as DADA2’s internal call to DECIPHER v.2.6.0 [39]. Further processing of 16S rRNA gene sequencing data was performed using Phyloseq [40]. Shotgun metagenomic sequences were demultiplexed, trimmed, and filtered using Trimmomatic v0.33 [41] with the following parameters: leading and trailing sequences of 10 bp, with a sliding window between 4 and 20 bp, and minimum length of 60 bp. Deconseq v0.4.3 on hsref38 was used to screen out any human DNA [42]. MetaPhlAn v2.0 [43] was then used to predict taxonomy down to the level of species. Functional pathways of the gut microbiome were inferred using HUMAnN2 by mapping unassembled sequencing reads to functionally annotated pangenomes [44]. The package prcomp v3.5.3 was used to calculate and plot the principal component analysis (PCA) of both the taxonomic and functional profiling, which were scaled during graphing (Fig. 2). The package ggbiplot was used to draw vectors corresponding to the contributions of the main taxa differentiating the “single intervention” (SI), “repeat intervention” (RI), and donor groups. A DPCOA plot with Euclidean distances was also generated through phyloseq.

## Results

### Taxonomic and functional pathway composition converge to a donor-like conformation after successful therapy

A multi-center trial of RBX2660 for recurrent CDI was conducted in 2013. Forty individuals were consented, and 31 patients completed the 6-month trial (Additional file 1: Fig. S1) [25]. Two patients had insufficient sampling frequency and were therefore excluded from our analysis leaving 29 individuals whose time courses of CDI symptoms, RBX2660 administration, and antibiotic receipt are shown in Fig. 1. Twelve patients did not experience a CDI recurrence after a single dose of the study drug (single intervention or SI group) while 17 experienced a recurrence between day 7 and day 60. The 17 patients with recurrent CDI received a repeat intervention with antibiotics and/or repeat RBX2660 (RI group; Fig. 1 Patient ID red text; median 15 days post initial RBX2660). Participants who received a second dose of RBX2660 were not necessarily pre-treated with antibiotics before as per study protocol (Additional file 1: Fig. S1) [25]. We first longitudinally determined the taxonomic composition of the gut microbiota after the first dose of RBX2660 [27] (Fig. 1, left panel). We used 16S rRNA gene sequencing analyzed via DADA2 [38] and computed weighted UniFrac distance from donor (DFD), which serves as a metric of engraftment [45]. After the first study treatment, the microbiota DFD shows a decreasing trend over time after treatment indicative of increased similarity with donor microbiota composition, but this differed by eventual treatment outcome (Fig. 2a). At time 0, there was no difference in median DFD between patients who responded to a single dose (SI) and those who received a repeat intervention (RI) for recurrent CDI (*p* > 0.05, Mann-Whitney *U* test). However, at day 7 after the first study treatment, microbiota DFD was significantly higher for individuals who eventually received repeat intervention after day 7 for recurrent CDI (Fig. 2b, median 0.31 vs. 0.22, Mann-Whitney *U* test, *p* < 0.05). The adoption of similar microbiota profiles to the donors by day 7 after the first study treatment is therefore significantly predictive of engraftment success of the initial therapy during the observation period. Although DFD appears to decrease for the RI group at day 60 (Fig. 2a), this observation is only based on the 4/17 individuals yet to experience CDI recurrence. These data demonstrate that in successful first treatments, the overall patient microbiota profile shifts quickly to resemble the donors after the study treatment. However, convergence is never absolute for these patients during the length of the study, with a mean DFD of 0.179 at 180 days after the first study treatment (Fig. 2a). This degree of engraftment is consistent with what has been reported previously for FMTs in the literature [22, 46].

**Fig. 3:**
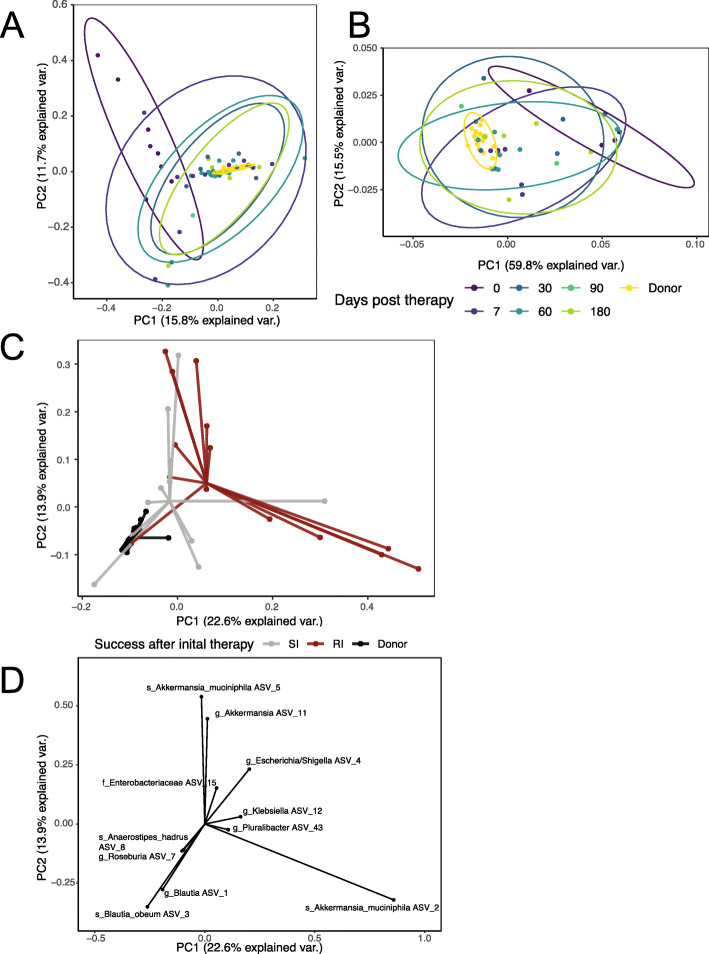
Taxonomy and microbial functional pathways converge after therapy receipt. **a**, **b** Principal component analysis (PCA) of patient microbiome taxonomic composition from 16S data (**a**) and of functional pathway abundances from whole metagenomic sequencing (**b**) in the SI group. Each colored dot represents an individual fecal sample after the first intervention with the circle representing 95% confidence interval with non-intersecting circles therefore statistically significant. Panel **a** shows 96 samples from all twelve patients with successful treatment and all four donors, while panel **b** shows 52 samples from eight successful patients and four donors (all of those who passed shotgun sequencing quality filters). **c** PCA from timepoint 7 samples after first study treatment only, colored by the SI or RI group (46 samples from all donors and all patients with day 7 samples; patient *N* = 25; donor *N* = 4). Each sample is connected to the centroid of its outcome group by a segment of the same color. **d** Taxonomy biplot shows the vectors of influence from taxa in distinguishing day 7 samples. The input samples, axes, and origin are the same as in **c**

We further investigated the impact of the microbiota-derived restoration therapy on the patient fecal microbiota using whole metagenomic shotgun sequencing with both taxonomic and functional profiling [27]. Each of the four donors contributed 2–8 samples for a total of 21 individual donor samples (Additional file 1: Fig. S2). The donor microbiota was dominated by Firmicutes, which is expected in healthy US adults [47], whereas the recipient microbiota prior to RBX2660 had increased abundance of Proteobacteria, which is a hallmark of antibiotic-disrupted microbiota [48, 49] (Additional file 1: Fig. S2). To explore this data, principle component analysis was performed. The PCAs in Fig. 3a and b were visualized for successful engraftments only (SI group), which revealed distinct microbiota communities between the donors and recipients at day 0 (as notated by nonoverlapping 95% confidence ellipses; Fig. 3a), but not thereafter. We next determined microbiome-wide functional pathways for the SI group as inferred using HUMAnN2 [44] (Fig. 3b). Similar to taxonomic composition, PCA of the diverse functional pathways found in these patients with successful treatments were significantly different (nonoverlapping 95% confidence ellipses; Fig. 3b) from those of the healthy donors only at the baseline timepoint. We also utilized Linear Discriminant Analysis with LEfSe to identify discriminatory features at 7 days indicative of receiving further intervention [50] (Additional file 1: Fig. S3). This analysis identified microbial pathways for membrane and biosynthetic processes were enriched in responders after the first dose. Individuals requiring re-intervention (RI group) had microbial functions enriched for flagella, pathogenesis, and ion binding. For a clearer picture of the changing trajectories over time, each of the HUMAnN2 pathways was plotted separately with each of the day 0 and day 7 pairs (Additional file 1: Fig. S4). These have been grouped by direction of change after treatment (i.e., whether a particular treatment group was enriched or depleted for a specific pathway after treatment). It is thus possible that certain microbial functions are restored after initial treatment (Additional file 1: Fig. S4), but patients still suffer CDI recurrence. Therefore, likelihood of successful treatment by RBX2660 is correlated with taxonomic and functional convergence to a more donor-like conformation.

### Key taxa discriminate those patients who require repeat intervention

To identify the specific microbial taxa correlated with treatment outcome, we utilized PCA to visualize differences in 16S rRNA gene-based taxonomic composition, as inferred by DADA2, between recipient day 7 samples stratified by eventual outcome as well as donor samples for comparison (Fig. 3c). The output from DADA2 is amplicon sequence variants (ASVs), which may differ by as few as 1 nucleotide and have been shown to improve specificity and sensitivity of organism identification [38, 51, 52]. The ASVs were numbered in order of overall prevalence within all samples for clarity. At day 7, patients who subsequently received a repeat intervention of either antibiotics or repeat RBX2660 therapy, the RI group, showed a significantly different taxonomic composition compared to either the SI group (Adonis, *p* = 0.028) or the donors (Adonis, *p* = 0.001) (Fig. 3c). The taxa identified by PCA driving the difference between the centroid positions included 25 ASVs above 5% importance and 11 above 10% importance (Fig. 3d). The taxonomy-labeled vectors influence the samples on Fig. 3c away from the origin in the direction indicated, so vectors pointing in the direction of the centroid of the donor represent important donor taxa, vectors in the direction of the SI group identify important features of success after initial therapy, and vectors pointing towards the centroid of the RI group identify features correlated with requiring additional treatment. In this PCA analysis of taxa at 7 days, the genera *Blautia* and *Roseburia* were most representative of the donors and success after initial therapy (SI), and ASVs representing members of the genera *Escherichia*/*Shigella*, *Klebsiella*, and *Pluralibacter* were most associated with likelihood of requiring repeat intervention (Fig. 3d). Three separate ASVs from the *Akkermansia* genus provided a large portion of the variation, and in some severely perturbed samples at day 0 and 7, *A. muciniphila* ASV 2 exceeded 40% of the entire microbial composition (Additional file 1: Fig. S5). After day 30, however, *A. muciniphila* ASV 2 often maintained a stable abundance of < 25% in the SI group, while abundance in the RI group was highly variable after re-intervention (Additional file 1: Fig. S5B). Replication of the PCA through phyloseq’s dpcoa function again showed *Akkermansia* contributing variation but not correlating with treatment outcome (Additional file 1: Fig. S6). Of note, *C. difficile* was not among the top indicators. Its corresponding ASV as well as *C. difficile* toxin genes, detected through custom ShortBRED markers (Additional file 1: Fig. S7A), were < 2% relative abundance in any sample and did not correlate with treatment outcome (Additional file 1: Fig. S7B).

**Fig. 4:**
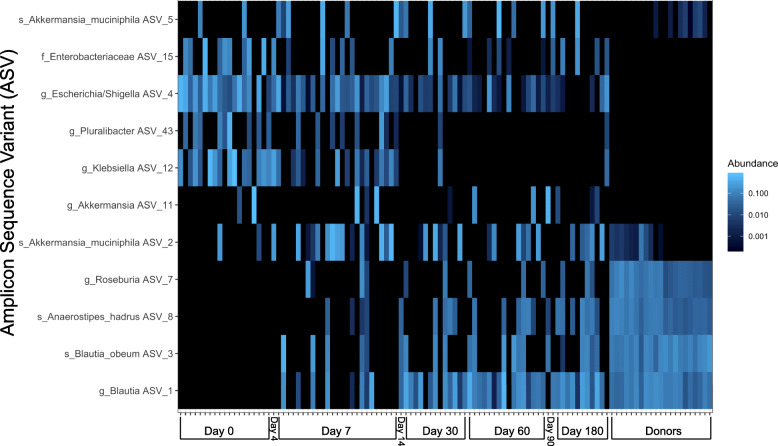
Taxa significantly associated with distance from the donor and successful response to RBX2660. A heatmap demonstrating the relative abundance over time after first RBX2660 is shown for donors and the SI group. These taxa are the top 11 identified by the PCA in Fig. 3d as significantly associated with successful treatment. Dark blue corresponds to 0.001% relative abundance with lighter blue 0.1% relative abundance. Each column represents a sample from a patient over time from left to right with donor samples at the right. *N* = 109 samples from 12 subjects from the SI group and 4 donors

Based on the findings from our PCA analysis, we temporally characterized the relative abundance of these 11 most discriminatory ASVs over time after the first treatment for the SI group (Fig. 4). For subjects who did not have CDI recurrence (the SI group), donor ASVs including *Roseburia* ASV7, *Blautia* ASVs 1 and 3, and *Anaerostipes* ASV8 were notably absent in day 0 specimens (Fig. 4). By day 7, these taxa increased in relative abundance, and by the end of the trial at day 180, their abundance was similar to the donor microbiome. Conversely, ASVs corresponding to Enterobacteriaceae, *Escherichia*, *Akkermansia*, and *Klebsiella* were abundant at time 0 for the recipients with their abundance declining over time. Thus, the taxa associated with a successful first RBX2660 treatment (Fig. 3d) begin to change relative abundance at 7 days after the first dose with continued adoption of a more donor-like conformation over the subsequent 180 days. At 30 days after the first study drug, 14/17 of the RI group had already suffered a CDI recurrence and received either a second FMT or antibiotics (Fig. 1). Thus, we cannot investigate whether the relative abundance differences at timepoints later than 7 days would also be associated with success. However, given the trends in relative abundance changes, it is likely that further adoption of a donor-like conformation would also be associated with success (Fig. 4). We have identified several taxa at 7 days after RBX2660 (Fig. 3d) whose presence and relative abundance changes during the first 180 days are associated with limiting CDI recurrence.

### Microbiota restoration concomitantly reduces antibiotic-resistant organisms and antibiotic resistance genes

**Fig. 5:**
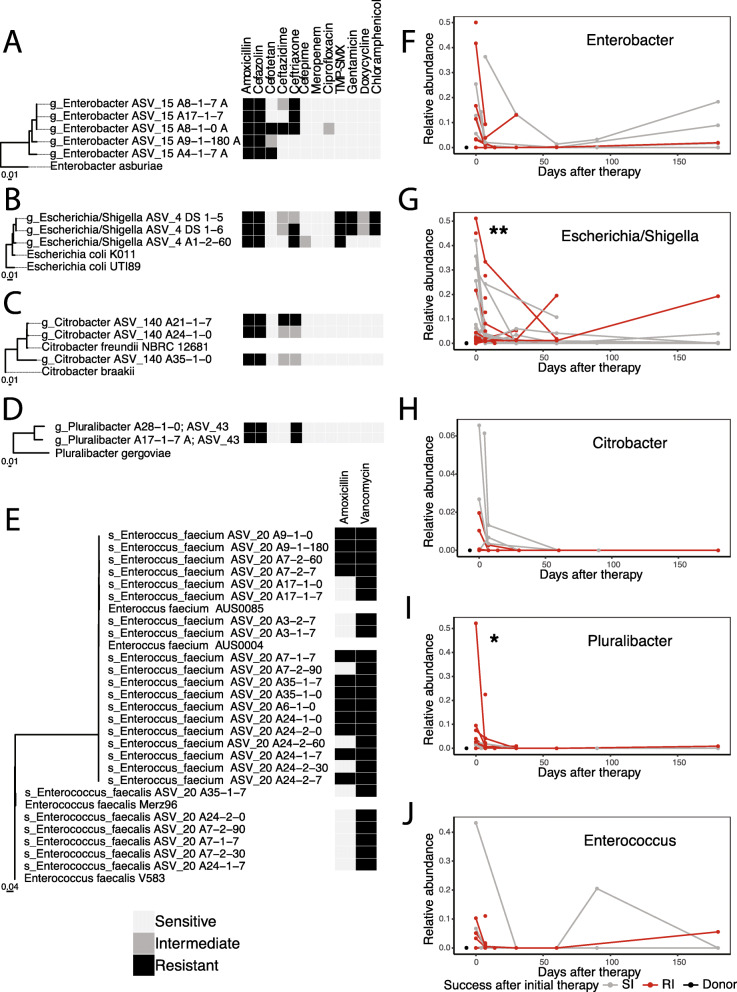
Antibiotic-resistant organisms cultured from patient and donor stools and the corresponding ASVs from species were tracked over time. **a**–**e** Antibiotic susceptibility profiles for each cultured organism from any sample from donor and patient with the corresponding phylogenetic tree. All breakpoints in antibiotic concentration were determined by CLSI 2016 criteria. Taxonomic labels are derived from DADA2 ASV assignments, with *Enterobacter* being further specified from family level based on metaphlan2 and MALDI-TOF taxonomy assignments. The designation A indicates recipient and D indicates donor. The following number indicates the study ID number followed by timepoint of isolation. A and E connote single colonies on separate plates. **f**–**j** Each of the ASVs corresponding to the species in **a**–**e** are shown in relative abundance over time in 131 fecal samples from 28 patients and 4 donors after the first study treatment. *p < 0.05, **p < 0.01 for relative abundance differences 7 days after therapy between SI and RI groups using Mann-Whitney *U* test. TMP-SMX, trimethoprim-sulfamethoxazole

Antibiotic resistance in the donor and recipient’s gut communities at any time was detected both by selective and differential culture and by annotation of ARGs from metagenomic shotgun sequencing data and assembled whole genome sequences of cultured isolates [35]. Selective and differential culture yielded 38 ARO isolates (5 *Enterobacter*, 3 *E. coli*, 3 *Citrobacter*, 2 *Pluralibacter*, 19 *Enterococcus faecium*, and 6 *Enterococcus faecalis*) identified by matrix-assisted laser desorption ionization time of flight mass spectrometry (MALDI-TOF MS) and confirmed via genomic analysis [27] (Additional file 2). Antibiotic susceptibility profiles (Fig. 5a–e) revealed resistance to 9 of 13 tested antibiotics across 5 genera, as measured by disk diffusion assay. A phylogenetic tree of all isolates was then created to demonstrate evolutionary relatedness and pruned to show each displayed isolate with the most closely related publicly available sequences.

*Enterobacter* (Fig. 5a), *Escherichia* (Fig. 5b), *Citrobacter* (Fig. 5c), and *Pluralibacter* (Fig. 5d) demonstrated phenotypic resistance to amoxicillin as well as 1st and 3rd generation cephalosporins. Multiple *E. coli* isolates additionally demonstrated resistance to gentamicin, doxycycline, and chloramphenicol (Fig. 5b). Importantly, given recent safety concerns regarding bacteremia caused by ESBL *E. coli* after FMT [53], we identified *E. coli* in a donor resistant to amoxicillin, cefazolin, and ceftriaxone indicative of ESBL production (Fig. 5b). Fortunately, neither patient receiving this product experienced an invasive infection from *E. coli* [25]. We also identified VR *Enterococcus faecalis* and *Enterococcus faecium* present in 8 patients throughout the course of the study (Fig. 5e). Annotation of the 41 assembled genomes with known ARGs through Resfinder detected 350 resistance genes predicting resistance to all major classes of antibiotics (Additional file 3). While the objective of this study was not to find or evaluate causal genotypes explaining empirical resistance, the AROs generally followed these rules: isolates with resistance to amoxicillin and cephalosporin antibiotics were typically associated with *bla* genes while *dfra1*, *aac/aadA1*, and *floR* corresponded to trimethoprim-sulfamethoxazole resistance, gentamicin resistance, and chloramphenicol resistance respectively (Additional file 3). There was no resistance to meropenem observed, and the single isolate with ciprofloxacin resistance did not have a known genomic marker associated. The cohort in this study harbored a substantial burden of ARGs and AROs, and we sought to track these species longitudinally via deeper metagenomic sampling.

The species corresponding to the 38 isolated AROs are common causes of healthcare-associated infection, are often multidrug resistant, and can participate in horizontal gene transfer between commensals and other pathogens within the gut and environment [54–56]. Because sequencing has the potential to be more sensitive at fecal detection of these organisms than bacterial culture [57], we sought to track these species within samples within our cohort. Accordingly, the ASVs associated with each ARO isolate that we identified were mapped by reconstructing rRNA genes in sequenced isolates, conducting in silico PCR to obtain 16S rRNA gene sequences, and annotating them with DADA2 (Fig. 5). ASVs corresponding to each cultured ARO (Fig. 5a–e) were tracked over time after first study drug (Fig. 5f–j). The relative abundance of the ASVs plotted in Fig. 5f–j represent multiple related strains with identical 16S rRNA sequences, which demonstrably contain all of the cultured AROs but can also represent susceptible sub-populations. However, with one exception, all of these ASVs were absent in the donors by both culture and metagenomics, allowing them to reliably measure the trajectory of the patient-associated ASVs. The ASV corresponding to the cultured *E. coli* isolates was found in one donor, and it was identified metagenomically in one donor (donor 1-1-DP) at 0.1% abundance. Accordingly, the two patients (patient IDs A2 and A26) receiving that product were not considered for eradication analysis for that ASV because we cannot distinguish between donor-derived *E. coli* and recipient-derived *E. coli*. Given the recent FDA alert of resistant *E. coli* infections after receipt of FMT [58], we confirmed that neither of these patients developed invasive infections from this organism. Excluding the donor-origin ARO and matched recipients, each other recipient sample that cultured an ARO was also positive by metagenomic sequencing, validating this mapping technique. Culture, however, detected AROs from these species in only 26/111 (23.4%) of the instances where that ASV was identified in the metagenomes. This may reflect differences in isolate viability in the stored fecal samples since dead cells will yield positive DNA-based detection. Alternatively, this may also reflect that ASVs for these species include both AROs and antibiotic susceptible forms of these bacteria. Thus, with this approach, the identified ASVs represent an upper-bound for detection of these potential AROs in the metagenomes.

After the first dose of RBX2660, the relative abundance of each isolate-based ASV diminished sharply (Fig. 5f–j). For each of these ASVs found in an individual’s earliest sample (*n* = 61 positive/130 total), if that ASV was undetectable in the patient’s last sample, it was considered eradicated. By this metric, 41/61 or 67% of these species were eradicated (Additional file 4). During the course of this study, 5 ASVs that were negative in both donors and in the patient’s earliest timepoints later became positive (3 *Enterobacter* ASV 15, 2 *Escherichia* ASV 4). These were considered either undetectably low abundance by metagenomic sequencing or environmentally acquired.

Despite the early decrease that we observed for ASVs corresponding to *Enterococcus*, *Escherichia*, and *Enterobacter*, some patients showed later variable increases in their abundance over time (Fig. 5f, g, j). Their respective eradication rates were 7/8 (87%), 9/22 (40%), and 7/10 (70%). However, *Pluralibacter* and *Citrobacte*r both remained at extremely low abundances (< 1% and .02%, respectively) following the initial depletion (Fig. 4h, i), with eradication rates of 5/6 (83%) and 7/7 (100%), respectively. Interestingly, despite the trend towards ARO decrease regardless of the outcome of first treatment, we found a significant difference in the relative abundance of both *Escherichia* (Fig. 5g, *p* < 0.01) and *Pluralibacter* (Fig. 5i, *p* < 0.05) ASVs between SI and RI groups at 7 days post-treatment. This finding corroborates the above analyses that sharp decreases in these genera may be associated with success whereas increased abundance at 7 days correlates with likelihood of failure of RBX2660. After 2nd study drug in the RI group, ASVs corresponding to *Escherichia*, *Citrobacter*, and *Enterococcus* did not decrease as dramatically with variable levels thereafter especially for *Escherichia* (Additional file 5: Fig. S8). ASV tracking in metagenomic samples allowed us to quantitatively assess the maximum possible abundance of these potential healthcare-associated infection-causing organisms. Importantly, this method of ASV tracking does not simultaneously measure phenotypic antibiotic resistance. As aforementioned, if patients carried closely related susceptible strains that were not found in the healthy donors, these could inflate the ASV totals. Nevertheless, this apparent rebound effect in ARO abundance that we identified via deeply sequencing isolates is especially important to consider when attempting to eradicate AROs completely from patient microbiomes via donor microbiota transfer. Furthermore, this method of tracking ASVs of predicted AROs in metagenomic samples is sensitive and robust to false negatives, and so it identifies frequent eradication and an overall decrease in AROs after microbiota-restoration therapy. We next proceeded to assess whether overall ARG content and identity decreased concomitantly with decreasing ARO abundance.

### Antibiotic resistance gene abundance decreases over time commensurate with adoption of donor microbiota

**Fig. 6:**
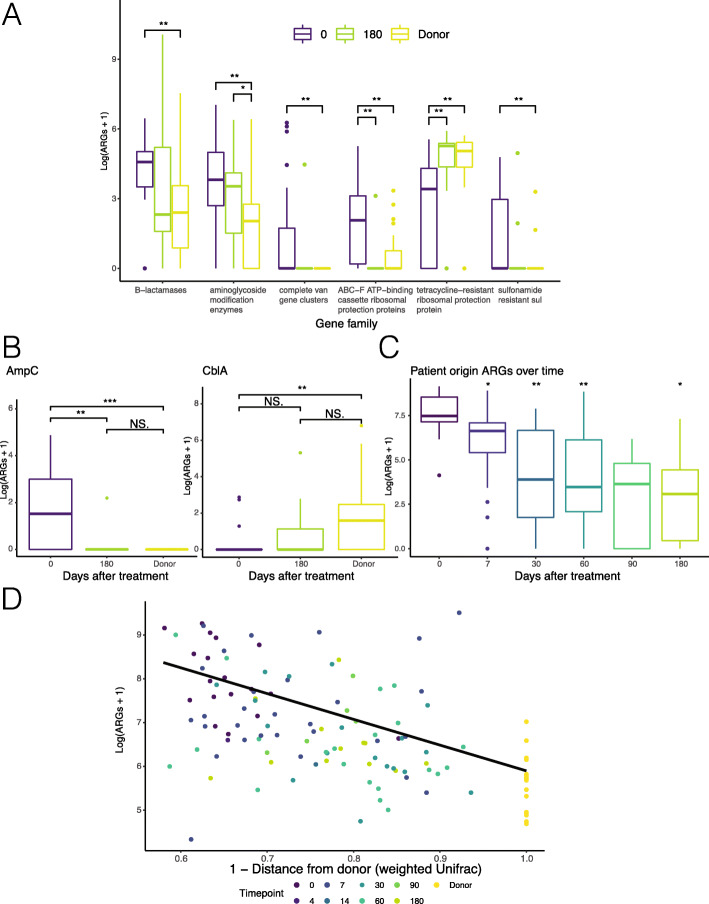
Antibiotic resistance gene abundance correlates with distance from the donor. **a** ARGs were quantified in metagenomic sequences (*N* = 21 patients and 4 donors) and summarized by mechanism. All ARG counts were transformed by log (ARG + 1) for visibility. **b** Two gene families within the β-lactamase class show opposite trajectories (*N* = 21 patients and 4 donors). **c** Patient-origin ARGs shown over time after RBX2660. **d** ARG abundance is plotted versus 1-(distance from donor) using weighted UniFrac. A generalized mixed effects log normal regression model of the formula ARGs ~ DFD + (1| PatientID) is shown, where DFD was significantly predictive of and correlated with ARG count compared to the null model (Chisq = 72.28, d.f.(full) = 1, *p* < 2.2 × 10^−16^). For **c** and **d**, all patients of both outcome groups were included for 153 total samples with patient *N* = 25 and donor *N* = 4. **a**–**c** Significance was determined by pairwise Wilcoxon with Benjamini-Hochberg correction. *p < 0.05; **p < 0.001

We annotated and quantified ARGs in each shotgun metagenome using ShortBRED with ARG markers built from the CARD database [37]. The most abundant ARG families (as determined by marker count per million reads) corresponding to major antibiotic classes were chosen for representation in Fig. 6a. For each gene family, the normalized gene abundance of all samples at timepoint 0 was compared to all samples from successful treatments (SI group) at timepoint 180 and to all donor samples (Fig. 6a, b). We chose to examine 180 days after intervention because prior research has shown microbiome recovery for healthy adults after antibiotic exposure [59]. For vancomycin, where multiple genes are required for functional resistance, the minimal complete cluster had to be present to be counted in this analysis. In every gene family, the abundance at timepoint 0 in patients was significantly different than in donors; and by timepoint 180, the abundance of that gene family in the patient had more closely approached that in the donor (Fig. 6a, b; pairwise Wilcoxon with Benjamini-Hochberg correction, *p* < 0.05). This was not always a decrease over time. Tetracycline resistance genes were most abundant within the donors and were gradually adopted by the recipients (Fig. 6a). Tetracycline resistance is commonly observed among healthy individuals given the inherent resistance of the most common microbial taxa [60, 61]. Within the β-lactamases, opposite effects were seen based on the origin of those genes, where AmpC-type β-lactamases were depleted while CblA genes were acquired and enriched (Fig. 6b). Altogether, the overall mean abundance of ARGs decreased over time (Fig. 6c), but not significantly in those patients requiring a repeated intervention nor after 2nd study drug (Additional file 5: Fig. S9). However, the best predictor of ARG carriage was not time from intervention but microbiota DFD. We observed a negative linear correlation between adoption of donor microbiota conformation as measured by 1-DFD closest to 1 (indicating increased donor similarity) and ARG carriage (Fig. 6d). The ARG burden therefore parallels the progress of RBX2660 engraftment as measured by 16S rRNA gene-based distance from donor (Fig. 2), showing a significant correlation in a linear mixed effects model (LR 17.68587, *p* < 0.0001). This overarching correlation holds true regardless of treatment status or origin of the ARGs. However, the strongest decrease was seen in patient-origin ARGs. There was no relationship between donor distance and the ARGs not present in baseline samples or donor (Additional file 5: Fig. S10). The rapidly changing patient microbiota samples had approximately 1 to 2 orders of magnitude greater variation than donor samples taken over the same time frame (Additional file 6). We therefore observed a strong ability of the donor microbiota to displace ARGs in the recipient, with the strength of this effect contingent on engraftment of the donor microbiota. Therefore, we have documented the ability of donor microbiota to reduce ARO species and ARG abundance as a collateral benefit of RBX2660 when successfully administered for prevention of recurrent CDI.

## Discussion

Microbiota transplantation has been utilized with great success to prevent recurrent CDI in many different trials and population subsets [18, 62, 63], albeit placebo-controlled clinical trial data [64] are still limited. However, suppression of blooms of *C. difficile* that cause CDI symptoms is not the same as pathogen eradication, nor does it necessarily operate by the same mechanism as would successful eradication or even suppression of ARO abundance. To characterize the effects and influences of this procedure on ARGs and carrier microbes, we have tracked bacterial taxonomic composition, microbial functional pathways, ARO colonization, and ARG abundance within the human gut microbiome for 6 months after the procedure. To aid in discerning directionality of association, we analyzed a cohort with variable engraftment, which can be leveraged as a dose-response relationship between treatment and effects from the gut microbiota. Engraftment of the donor microbiota was determined via 16S rRNA gene sequencing of the patient and donor, and calculation of weighted UniFrac distance from donor (DFD) 7 days after product delivery. Patients with successful RBX2660 treatment were distinguishable from those who required further treatment based on their microbiome taxonomic and functional composition 1 week after treatment (Figs. 2, 3, and 4). Furthermore, we have identified key taxa associated with CDI treatment outcomes. Specifically, members of the Lachnospiraciae family (*Blautia* spp., *Roseburia*, and *Anaerostipes*) were correlated with success whereas high abundance of Proteobacteria (*Escherichia*, *Klebsiella*, and *Pluralibacter*) at day 7 was associated with the necessity of additional treatment in this cohort (Figs. 3 and 4).

The taxonomic restructuring was dominated by reduction of *Enterobacteriaceae* initially after therapy (Fig. 5). These species are known to bloom after use of antibiotics, and their presence in high numbers at baseline is expected in this cohort. Our observation that increased pathways dedicated to flagellin and motility are associated with treatment failure is consistent with the knowledge that Proteobacteria such as *E. coli* and *Klebsiella* are often flagellated and motile (Additional file 1: Fig. S3). In treatment of this dysbiosis, the Enterobacterales give way to Firmicutes, particularly *Lachnospiraciae*. It has been previously demonstrated that *Blautia obeum* expresses bile salt hydrolases that have been shown to suppress *C. difficile* germination in animal models [65]. Another important player is *Akkermansia muciniphila*, which has been inversely associated with mucosal membrane pathology in multiple gastrointestinal disorders [66, 67]. Specifically, *Akkermansia* spp. are important for mucin degradation, and their relative absence is associated with insulin resistance, diabetes, and inflammatory bowel disease in both human cohorts and animal models (reviewed in [68]). In this cohort, healthy donors and patients with successful donor engraftments contain stable lower levels of *Akkermansia muciniphila*, while pre-treatment samples and potentially dysbiotic microbiomes (high microbiota DFD) have widely varying levels. We speculate that in this instance, *Akkermansia* is a surrogate for microbiome health, a hypothesis that would require further validation in other cohorts and models. *C. difficile* itself was not among the microbial taxa with strong associations to CDI symptoms. This could be because sampling often occurred days or weeks apart from reported CDI symptoms (Fig. 1). Restoration of the microbiome to a healthy configuration as quantified by low microbiota DFD is the best microbiome correlate with symptom reduction that we identified. That sporulation was a positive predictor of success is intriguing because it may suggest that colonization resistance to *C. difficile* may be enhanced by other sporulating bacteria, as has been speculated in development of more defined probiotic cocktails for treatment of CDI [20, 69–71]. In this sample set of 29 patients, those whose microbiota DFD reduced by less than 20% by day 7 were more likely to require further treatment (Fig. 2b). In support of this, increased abundance of Bacteroidia and Clostridia 7 days after RBX2660 correlated with a recurrence-free interval in a prior study [46]. Thus, our study identifies several taxa correlated with FMT success or failure that can be evaluated in larger, placebo-controlled studies.

The overall convergence of patient microbiomes with donors in both taxonomy and microbial functional pathways was concordant with a competitive mechanism of donor microbial engraftment similar to that identified by Smillie et al. [22]. Previous literature has demonstrated that the efficacy of FMT on CDI symptoms depends on engraftment efficiency [22], and we corroborate that here with this investigational microbiota therapeutic. Another intriguing finding supported by this study is that over the follow-up period after treatment, patients can and do acquire both taxa and ARGs that were not present at baseline or in the donor [22]. In our study, those taxa and ARGs (Additional file 5: Fig. S10) did not show any engraftment completion-related trends, so they behaved differently than either patient or donor origin. These taxa and genes could either be undetectably low at baseline and in the donor, or they could come from the environment. This highlights the potential importance of the patient’s environment after FMT for determining and maintaining a healthy gut microbiota composition.

We hypothesized that microbiota restoration would be accompanied by a decrease in ARO and ARG carriage. This hypothesis was indeed true with greatest impact when the recipient’s microbiota adopted a conformation similar to the donor at 7 days after therapy (Figs. 5, 6). This observation underscores the dramatic and immediate restructuring of the microbial ecology of the gut following a successful FMT in all three levels of taxonomy, microbial metabolism, and ARG carriage. In order to better address the impact of the FMT intervention and fates of ARO and ARG thereafter, a placebo group should be included in future studies [64]. Post-FMT microbiota composition is dictated largely by abundance in the donor and recipient. ARG abundance was higher before treatment, and after RBX2660 was directly correlated with DFD. That is, better engraftment of the FMT leads to a more donor-like conformation and reduced ARG abundance. For AROs, we did not observe this universally. We instead found that for VRE and ARO Enterobacteriaceae, relative abundances as high as 40% were reduced by donor product. Thus, outcome of FMT is not exclusively based on taxonomic abundance of either the donor or recipient prior to administration.

The effects of microbiota restoration on AROs is a topic of hope and contention in the literature [72]. We add to that body of literature ASV tracking of potential AR pathogens and longitudinal relative abundance from well-sampled 16S rRNA gene sequencing, which is among the most sensitive detection methods available. The eradication rate from all cultured ARO taxa (67%) was within the wide range of 37.5–87.5% expected based on FMT for any condition [72]. Between genera of AROs in this study, eradication rates varied from 40% in *Escherichia* to 100% in *Citrobacter.* The slight rebound of relative abundance found in 3/5 of tracked ASVs at 180 days would potentially be undetectable by culture. Yet this rebound could be clinically important, as further antibiotic selection on a patient with incomplete eradication could be riskier than on a patient who has been successfully decolonized. Importantly, while we only cultured ARO *E. coli* from one donor product (compared to 40 other ARO isolates from patients), we did metagenomically detect this ASV in both of the recipients of this donor product. This finding is especially important after two individuals suffered bacteremia from ESBL producing *E. coli* present in donor FMT resulting in the FDA requiring donor screening questionnaires and MDRO testing of donor stool [53, 58]. This finding highlights the importance of screening donor products for ARO isolates to reduce their risk of acquisition by recipients. Establishing appropriate detection thresholds for bacteria targeted for donation, eradication, and replacement is therefore critically important in these studies.

This study presents taxa and microbial functional pathways that require larger datasets and further validation prior to incorporation into clinical practice. Furthermore, sample storage conditions and freeze-thaw cycles have been shown to decrease certain taxa, especially Bacteroidetes [73], which is an inherent limitation of performing microbiome analyses on archived fecal samples. Additionally, this study did not include a placebo group that did not receive FMT in order to characterize the natural history of ARG and ARO decrease after finishing a course of antibiotics. The abundance of ARGs in the gut microbiome was even more clearly responsive to treatment in a strong inverse relationship to engraftment, as measured by DFD. It is reasonable that both shedding and transmission of microbes reduce when they are present at lower abundance in the gut, but the epidemiology of this remains unquantified. The high initial burden of ARGs and AROs in CDI patients [23], along with growing incidence (and/or reporting) of CDI [74], is an important additional motivation for global surveillance of AR and development of methods to combat its spread. Finally, most microbiome analyses to date have focused on metagenomic sequencing of bacteria in stool samples, but emerging research suggests that viruses, prokaryotes, and small molecules can also meaningfully impact health and disease [75, 76]. Therefore, future studies should consider a multi-omics and multi-kingdom approach to better predict outcomes after FMT to both restore microbiome health and limit ARG and ARO carriage.

## Conclusions

We have demonstrated here that in addition to the important prevention of recurrent CDI, when the donor microbiome optimally engrafts after microbiota-restoration therapy, ARG and ARO abundance in the recipient gut microbiomes substantially decrease. Abundance of ASVs corresponding to species that are potentially multidrug resistant in baseline samples was immediately reduced and often to undetectable levels, but a late rebound for some patients indicates incomplete eradication. Further studies are needed to quantify epidemiological benefits such as decreased transmission to other people and the environment. Thus, RBX2660 and microbial therapeutics in general represent an effective method to alter the gut community composition together with all its metabolic and potentially pathologic attributes. The abundance of ARGs and AROs can potentially be lastingly reduced with this method, making it a promising tool in combating the global threat of antibiotic resistance.

## Supplementary Information

**Additional file 1: Fig. S1.** Study protocol for Phase II clinical trial NCT01925417 adapted from reference [25]. Samples specifically used for this study depicted in Fig. 1. **Fig. S2.** The relative abundance of bacterial phyla in all patients are shown at day 0 (panel A) and in all donor samples from the 4 donors (panel B). The patient IDs are marked in gray if symptoms resolved in a single dose of the study drug and in red if they required repeat intervention. A) Patient ID is listed after the letter A on the x axis with relative abundance of each phylum in stacked bar chart format on the y-axis. B) Donor samples are named as donor number.samplenumber followed by DS for donor substance. **Fig. S3.** linear discriminant analysis compares functional pathway abundance, as annotated by HUMAnN2 and visualized with LEfSe, at day 7 between the two outcome groups. In red are pathways more enriched in the reintervention (RI) group, while those patients who recovered after a single treatment had a significantly higher abundance of the pathways in green. **Fig. S4**. Shown are the HUMAnN2 functional pathway abundances for all patients with both day 0 and day 7 samples. **Fig. S5.** The relative abundance of *Akkermansia muciniphila* ASV 2 is shown over time stratified by outcome. A) The patient IDs are marked in gray if symptoms resolved in a single dose of the study drug, in red if they required repeat intervention, and black if they come from the donor. B) *Akkermansia muciniphila* ASV2 abundance after re-intervention. **Fig. S6.** The PCA analysis from Main Fig. 2 is reproduced here via the dual principal component function of phyloseq, which uses Euclidean distances. The overall taxonomic composition is graphed in Panel A, while Panel B shows the directionality of the influence of individual taxa upon those samples. **Fig. S7. A)** The relative abundance of *Clostridioides difficile* is tracked here using species-specific toxin genes detected in metagenomic sequences via ShortBRED. The gene count was normalized to the number of metagenomic reads and expressed in terms of copies per metagenome. **B)** The relative abundance for the ASV corresponding to *C. difficile* is shown in the bottom panel. The patient IDs are marked in gray if symptoms resolved in a single dose of the study drug and in red if they required repeat intervention.

**Additional file 2.** This table shows cultured isolates, their associated ASVs, their taxonomy assignments according to DADA2 and MALDI-TOF, and antibiotic sensitivity results in terms of their clearance zone sizes and the interpretations of sensitive, intermediate, or resistant. Final taxonomy assignments were confirmed by genomic alignments with type strains.

**Additional file 3.** All genomic resistance gene annotations from Resfinder for all MDRO isolates are listed here, with their specific genomic location, predicted phenotype, and % identity to reference genes.

**Additional file 4.** The eradication status of ARO found in each patient is summarized here. If the first sample (notated as Patient ID – Dose number – Days from previous dose) was positive according to the ASV quantified by DADA2 and the last sample from the same patient was negative, the status for that ASV is shown as negative. Acquired means it was first negative (in both patient and donor) and later positive, absent means all samples were negative, masked means the donor was positive, and insufficient samples means the first and last sample were the same.

**Additional file 5 Fig. S8.** Relative abundance of ASVs corresponding to A) *Enterobacter*, B) *Escherichia*, C) *Citrobacter*, and D) *Enterococcus* tracked temporally after second dose of RBX2660 in the RI group. **Fig. S9.** Patient origin ARGs over time after A) first RBX2660 and B) second RBX2660 in the RI group. All comparisons non-significant as determined by pairwise Wilcoxon with Benjamini Hochberg correction. *n* = 17 total patients with A) 27 and B) 45 samples. **Fig. S10.** Abundance of resistance genes in each metagenomic sample compared to their DFD for resistance genes that were not detected in patients’ day 0 samples or in the donors. For these genes, their abundance and the distance from donor are uncorrelated.

**Additional file 6.** The two tables show the total ARG hits in RPKM for donors and patients at each sample collection timepoint, as well as the standard deviation and variance over time.

## Data Availability

The 16s rRNA and raw genomic, metagenomic DNA dataset generated/and or analyzed during the current study are available in the NCBI repository under BioProject PRJNA674880: https://www.ncbi.nlm.nih.gov/bioproject/674880 [27]. The genome isolate assemblies are available in the NCBI repository under BioProject PRJNA693986: https://www.ncbi.nlm.nih.gov/bioproject/693986 [35].

## Abbreviations

CDI: *Clostridioides difficile* infection
AR: Antibiotic resistant/resistance
AROs: Antibiotic-resistant organisms
ARGs: Antibiotic resistance genes
VRE: Vancomycin-resistant *Enterococci*
FMT: Fecal microbiota transplantation
MRSA: Methicillin-resistant *Staphylococcus aureus*
ESBL: Extended-spectrum beta lactamase
ASVs: Amplicon sequence variants
PCA: Principal component analysis
DFD: Distance from donor
RI: Repeat intervention
SI: Single intervention

## References

[1] O'Neill J (2016). Tackling drug-resistant infections globally: final report and recommendations.

[2] Klein EY, Van Boeckel TP, Martinez EM, Pant S, Gandra S, Levin SA (2018). Global increase and geographic convergence in antibiotic consumption between 2000 and 2015. Proc Natl Acad Sci U S A.

[3] Pehrsson EC, Tsukayama P, Patel S, Mejía-Bautista M, Sosa-Soto G, Navarrete KM (2016). Interconnected microbiomes and resistomes in low-income human habitats. Nature..

[4] Costelloe C, Metcalfe C, Lovering A, Mant D, Hay AD (2010). Effect of antibiotic prescribing in primary care on antimicrobial resistance in individual patients: systematic review and meta-analysis. BMJ..

[5] Wang J, Foxman B, Mody L, Snitkin ES (2017). Network of microbial and antibiotic interactions drive colonization and infection with multidrug-resistant organisms. Proc Natl Acad Sci U S A.

[6] Langdon A, Crook N, Dantas G (2016). The effects of antibiotics on the microbiome throughout development and alternative approaches for therapeutic modulation. Genome Med.

[7] Chatterjee A, Modarai M, Naylor NR, Boyd SE, Atun R, Barlow J (2018). Quantifying drivers of antibiotic resistance in humans: a systematic review. Lancet Infect Dis.

[8] Buffie CG, Pamer EG (2013). Microbiota-mediated colonization resistance against intestinal pathogens. Nat Rev Immunol.

[9] Chilton CH, Pickering DS, Freeman J (2018). Microbiologic factors affecting Clostridium difficile recurrence. Clin Microbiol Infect.

[10] Lessa FC, Winston LG, McDonald LC, Emerging Infections Program CST (2015). Burden of Clostridium difficile infection in the United States. N Engl J Med.

[11] Shah D, Dang MD, Hasbun R, Koo HL, Jiang ZD, DuPont HL (2010). Clostridium difficile infection: update on emerging antibiotic treatment options and antibiotic resistance. Expert Rev Anti-Infect Ther.

[12] Weingarden A, Gonzalez A, Vazquez-Baeza Y, Weiss S, Humphry G, Berg-Lyons D (2015). Dynamic changes in short- and long-term bacterial composition following fecal microbiota transplantation for recurrent Clostridium difficile infection. Microbiome..

[13] Youngster I, Sauk J, Pindar C, Wilson RG, Kaplan JL, Smith MB (2014). Fecal microbiota transplant for relapsing Clostridium difficile infection using a frozen inoculum from unrelated donors: a randomized, open-label, controlled pilot study. Clin Infect Dis.

[14] Al-Nassir WN, Sethi AK, Li Y, Pultz MJ, Riggs MM, Donskey CJ (2008). Both oral metronidazole and oral vancomycin promote persistent overgrowth of vancomycin-resistant enterococci during treatment of Clostridium difficile-associated disease. Antimicrob Agents Chemother.

[15] Roghmann MC, McCarter RJ, Brewrink J, Cross AS, Morris JG (1997). Clostridium difficile infection is a risk factor for bacteremia due to vancomycin-resistant enterococci (VRE) in VRE-colonized patients with acute leukemia. Clin Infect Dis.

[16] Prematunge C, MacDougall C, Johnstone J, Adomako K, Lam F, Robertson J (2016). VRE and VSE bacteremia outcomes in the era of effective VRE therapy: a systematic review and meta-analysis. Infect Control Hosp Epidemiol.

[17] Cammarota G, Ianiro G, Gasbarrini A (2014). Fecal microbiota transplantation for the treatment of Clostridium difficile infection: a systematic review. J Clin Gastroenterol.

[18] Iqbal U, Anwar H, Karim MA (2018). Safety and efficacy of encapsulated fecal microbiota transplantation for recurrent Clostridium difficile infection: a systematic review. Eur J Gastroenterol Hepatol.

[19] Dubberke ER, Lee CH, Orenstein R, Khanna S, Hecht G, Gerding DN (2018). Results from a randomized, placebo-controlled clinical trial of a RBX2660-a microbiota-based drug for the prevention of recurrent Clostridium difficile infection. Clin Infect Dis.

[20] Khanna S, Pardi DS, Kelly CR, Kraft CS, Dhere T, Henn MR (2016). A novel microbiome therapeutic increases gut microbial diversity and prevents recurrent Clostridium difficile infection. J Infect Dis.

[21] Suez J, Zmora N, Zilberman-Schapira G, Mor U, Dori-Bachash M, Bashiardes S (2018). Post-antibiotic gut mucosal microbiome reconstitution is impaired by probiotics and improved by autologous FMT. Cell..

[22] Smillie CS, Sauk J, Gevers D, Friedman J, Sung J, Youngster I (2018). Strain tracking reveals the determinants of bacterial engraftment in the human gut following fecal microbiota transplantation. Cell Host Microbe.

[23] Millan B, Park H, Hotte N, Mathieu O, Burguiere P, Tompkins TA (2016). Fecal microbial transplants reduce antibiotic-resistant genes in patients with recurrent Clostridium difficile infection. Clin Infect Dis.

[24] Singh R, de Groot PF, Geerlings SE, Hodiamont CJ, Belzer C, Berge I (2018). Fecal microbiota transplantation against intestinal colonization by extended spectrum beta-lactamase producing Enterobacteriaceae: a proof of principle study. BMC Res Notes.

[25] Orenstein R, Dubberke E, Hardi R, Ray A, Mullane K, Pardi DS (2016). Safety and durability of RBX2660 (microbiota suspension) for recurrent *Clostridium difficile* infection: results of the PUNCH CD study. Clin Infect Dis.

[26] Ray A, Jones C (2016). Does the donor matter? Donor vs patient effects in the outcome of a next-generation microbiota-based drug trial for recurrent Clostridium difficile infection. Future Microbiol.

[27] Langdon A, Schwartz DJ, Bulow C, Sun X, Hink T, Reske KA, et al. Dataset for microbiota restoration reduces antibiotic resistant bacteria gut colonization in patients with recurrent Clostridioides difficile infection from the open-label PUNCH CD study. NCBI Biorepository 2020. 10.1186/s13073-021-00843-9 PMC7888090 33593430

[28] CLSI, editor. Performance standards for antimicrobial susceptibility testing. 26th ed. ed: Clinical and Laboratory Standards Institute; 2016.

[29] Gurevich A, Saveliev V, Vyahhi N, Tesler G (2013). QUAST: quality assessment tool for genome assemblies. Bioinformatics.

[30] Zankari E, Hasman H, Cosentino S, Vestergaard M, Rasmussen S, Lund O (2012). Identification of acquired antimicrobial resistance genes. J Antimicrob Chemother.

[31] Seemann T (2014). Prokka: rapid prokaryotic genome annotation. Bioinformatics.

[32] Page AJ, Cummins CA, Hunt M, Wong VK, Reuter S, Holden MT (2015). Roary: rapid large-scale prokaryote pan genome analysis. Bioinformatics.

[33] Stamatakis A (2014). RAxML version 8: a tool for phylogenetic analysis and post-analysis of large phylogenies. Bioinformatics.

[34] Seah BKB, Schwaha T, Volland JM, Huettel B, Dubilier N, Gruber-Vodicka HR. Specificity in diversity: single origin of a widespread ciliate-bacteria symbiosis. Proc Biol Sci. 2017;284(1858):1–9. 10.1098/rspb.2017.0764 PMC5524500 28701560

[35] Langdon A, Schwartz DJ, Bulow C, Sun X, Hink T, Reske KA, et al. Dataset for bacterial isolate assemblies for microbiota restoration reduces antibiotic resistant bacteria gut colonization in patients with recurrent Clostridioides difficile infection from the open-label PUNCH CD study. NCBI Biorepository 2020. https://www.ncbi.nlm.nih.gov/bioproject/?term=693986 10.1186/s13073-021-00843-9 PMC7888090 33593430

[36] Schloss PD, Westcott SL, Ryabin T, Hall JR, Hartmann M, Hollister EB (2009). Introducing mothur: open-source, platform-independent, community-supported software for describing and comparing microbial communities. Appl Environ Microbiol.

[37] Kaminski J, Gibson MK, Franzosa EA, Segata N, Dantas G, Huttenhower C (2015). High-specificity targeted functional profiling in microbial communities with ShortBRED. PLoS Comput Biol.

[38] Callahan BJ, McMurdie PJ, Rosen MJ, Han AW, Johnson AJA, Holmes SP (2016). DADA2: high-resolution sample inference from Illumina amplicon data. Nature Methods.

[39] Wright ES (2015). DECIPHER: harnessing local sequence context to improve protein multiple sequence alignment. BMC Bioinformatics.

[40] McMurdie PJ, Holmes S (2013). phyloseq: an R package for reproducible interactive analysis and graphics of microbiome census data. Plos One.

[41] Bolger AM, Lohse M, Usadel B (2014). Trimmomatic: a flexible trimmer for Illumina sequence data. Bioinformatics.

[42] Schmieder R, Edwards R (2011). Fast identification and removal of sequence contamination from genomic and metagenomic datasets. Plos One.

[43] Truong DT, Franzosa EA, Tickle TL, Scholz M, Weingart G, Pasolli E (2015). MetaPhlAn2 for enhanced metagenomic taxonomic profiling. Nat Methods.

[44] Franzosa EA, McIver LJ, Rahnavard G, Thompson LR, Schirmer M, Weingart G (2018). Species-level functional profiling of metagenomes and metatranscriptomes. Nat Methods.

[45] Le Roy T, Debedat J, Marquet F, Da-Cunha C, Ichou F, Guerre-Millo M (2018). Comparative evaluation of microbiota engraftment following fecal microbiota transfer in mice models: age, kinetic and microbial status matter. Front Microbiol.

[46] Blount KF, Shannon WD, Deych E, Jones C. Restoration of bacterial microbiome composition and diversity among treatment responders in a phase 2 trial of Rbx2660—an investigational microbiome restoration therapeutic. Open Forum Infect Dis. 2019:6(4)ofz095:1–10. 10.1093/ofid/ofz095 PMC6475591 31024971

[47] Consortium THMP, Huttenhower C, Gevers D, Knight R, Abubucker S, Badger JH (2012). Structure, function and diversity of the healthy human microbiome. Nature..

[48] Citron DM, Tyrrell KL, Dale SE, Chesnel L, Goldstein EJ (2016). Impact of surotomycin on the gut microbiota of healthy volunteers in a phase 1 clinical trial. Antimicrob Agents Chemother.

[49] Reuland EA, Sonder GJ, Stolte I, Al Naiemi N, Koek A, Linde GB (2016). Travel to Asia and traveller’s diarrhoea with antibiotic treatment are independent risk factors for acquiring ciprofloxacin-resistant and extended spectrum beta-lactamase-producing Enterobacteriaceae-a prospective cohort study. Clin Microbiol Infect.

[50] Segata N, Izard J, Waldron L, Gevers D, Miropolsky L, Garrett WS (2011). Metagenomic biomarker discovery and explanation. Genome Biol.

[51] Callahan BJ, McMurdie PJ, Holmes SP (2017). Exact sequence variants should replace operational taxonomic units in marker-gene data analysis. ISME J.

[52] Mukherjee C, Beall CJ, Griffen AL, Leys EJ (2018). High-resolution ISR amplicon sequencing reveals personalized oral microbiome. Microbiome..

[53] DeFilipp Z, Bloom PP, Torres Soto M, Mansour MK, Sater MRA, Huntley MH (2019). Drug-resistant E. coli bacteremia transmitted by fecal microbiota transplant. N Engl J Med.

[54] Babady NE. Hospital-associated infections. Microbiol Spectr. 2016;4(3):1–22. 10.1128/microbiolspec.DMIH2-0003-2015 27337459

[55] Naiemi NA, Duim B, Savelkoul PH, Spanjaard L, de Jonge E, Bart A (2005). Widespread transfer of resistance genes between bacterial species in an intensive care unit: implications for hospital epidemiology. J Clin Microbiol.

[56] Forsberg KJ, Reyes A, Wang B, Selleck EM, Sommer MO, Dantas G (2012). The shared antibiotic resistome of soil bacteria and human pathogens. Science.

[57] Andersen H, Connolly N, Bangar H, Staat M, Mortensen J, Deburger B (2016). Use of shotgun metagenome sequencing to detect fecal colonization with multidrug-resistant bacteria in children. J Clin Microbiol.

[58] Administration FaD. Information pertaining to additional safety protections regarding use of fecal microbiota for transplantation – screening and testing of stool donors for multi-drug resistant organisms FDA.gov2019 [updated 06/18/2019.

[59] Palleja A, Mikkelsen KH, Forslund SK, Kashani A, Allin KH, Nielsen T (2018). Recovery of gut microbiota of healthy adults following antibiotic exposure. Nat Microbiol.

[60] Buelow E, Bello Gonzalez TDJ, Fuentes S, de Steenhuijsen Piters WAA, Lahti L, Bayjanov JR (2017). Comparative gut microbiota and resistome profiling of intensive care patients receiving selective digestive tract decontamination and healthy subjects. Microbiome..

[61] Feng J, Li B, Jiang X, Yang Y, Wells GF, Zhang T (2018). Antibiotic resistome in a large-scale healthy human gut microbiota deciphered by metagenomic and network analyses. Environ Microbiol.

[62] Tariq R, Pardi DS, Bartlett MG, Khanna S (2019). Low cure rates in controlled trials of fecal microbiota transplantation for recurrent Clostridium difficile infection: a systematic review and meta-analysis. Clin Infect Dis.

[63] Khan MY, Dirweesh A, Khurshid T, Siddiqui WJ (2018). Comparing fecal microbiota transplantation to standard-of-care treatment for recurrent Clostridium difficile infection: a systematic review and meta-analysis. Eur J Gastroenterol Hepatol.

[64] Kwak S, Choi J, Hink T, Reske KA, Blount K, Jones C (2020). Impact of investigational microbiota therapeutic RBX2660 on the gut microbiome and resistome revealed by a placebo-controlled clinical trial. Microbiome..

[65] Mullish BH, McDonald JAK, Pechlivanis A, Allegretti JR, Kao D, Barker GF, et al. Microbial bile salt hydrolases mediate the efficacy of faecal microbiota transplant in the treatment of recurrent Clostridioides difficile infection. Gut. 2019;68(10):1791–800. 10.1136/gutjnl-2018-317842 PMC6839797 30816855

[66] Png CW, Linden SK, Gilshenan KS, Zoetendal EG, McSweeney CS, Sly LI (2010). Mucolytic bacteria with increased prevalence in IBD mucosa augment in vitro utilization of mucin by other bacteria. Am J Gastroenterol.

[67] Dao MC, Everard A, Aron-Wisnewsky J, Sokolovska N, Prifti E, Verger EO (2016). Akkermansia muciniphila and improved metabolic health during a dietary intervention in obesity: relationship with gut microbiome richness and ecology. Gut..

[68] Derrien M, Belzer C, de Vos WM (2017). Akkermansia muciniphila and its role in regulating host functions. Microb Pathog.

[69] Ford C HM, Bryant J, Diao L, Wortman J, Tomlinson A, Litcofsky K, Bernardo P, McGovern B, Aunins JG, Cook DN, Trucksis M. 1641. Treatment of recurrent Clostridium difficile infection with SER-109 reduces gastrointestinal carriage of antimicrobial resistance genes. Open Forum Infect Dis. 2018;5:S44-S47.

[70] Browne HP, Forster SC, Anonye BO, Kumar N, Neville BA, Stares MD (2016). Culturing of ‘unculturable’ human microbiota reveals novel taxa and extensive sporulation. Nature..

[71] Lawley TD, Clare S, Walker AW, Goulding D, Stabler RA, Croucher N (2009). Antibiotic treatment of clostridium difficile carrier mice triggers a supershedder state, spore-mediated transmission, and severe disease in immunocompromised hosts. Infect Immun.

[72] Saha S, Tariq R, Tosh PK, Pardi DS, Khanna S (2019). Faecal microbiota transplantation for eradicating carriage of multidrug-resistant organisms: a systematic review. Clin Microbiol Infect.

[73] McKain N, Genc B, Snelling TJ, Wallace RJ (2013). Differential recovery of bacterial and archaeal 16S rRNA genes from ruminal digesta in response to glycerol as cryoprotectant. J Microbiol Methods.

[74] Ma GK, Brensinger CM, Wu Q, Lewis JD (2017). Increasing incidence of multiply recurrent Clostridium difficile infection in the United States: a cohort study. Ann Intern Med.

[75] Lloyd-Price J, Arze C, Ananthakrishnan AN, Schirmer M, Avila-Pacheco J, Poon TW (2019). Multi-omics of the gut microbial ecosystem in inflammatory bowel diseases. Nature..

[76] Lim ES, Zhou Y, Zhao G, Bauer IK, Droit L, Ndao IM (2015). Early life dynamics of the human gut virome and bacterial microbiome in infants. Nat Med.

