# Supplementary material for: Microbiota restoration reduces antibiotic-resistant bacteria gut colonization in patients with recurrent Clostridioides difficile infection from the open-label PUNCH CD study
Source: Genome Med. 2021 Feb 16;13:28. doi: 10.1186/s13073-021-00843-9 (PMC7888090; doi:10.1186/s13073-021-00843-9)
Supplement: Supplementary file 1 — Additional file 1: Fig. S1. Study protocol for Phase II clinical trial NCT01925417 adapted from reference [25]. Samples specifically used for this study depicted in Fig. 1. Fig. S2. The relative abundance of bacterial phyla in all patients are shown at day 0 (panel A) and in all donor samples from the 4 donors (panel B). The patient IDs are marked in gray if symptoms resolved in a single dose of the study drug and in red if they required repeat intervention. A) Patient ID is listed after the letter A on the x axis with relative abundance of each phylum in stacked bar chart format on the y-axis. B) Donor samples are named as donor number.samplenumber followed by DS for donor substance. Fig. S3. linear discriminant analysis compares functional pathway abundance, as annotated by HUMAnN2 and visualized with LEfSe, at day 7 between the two outcome groups. In red are pathways more enriched in the reintervention (RI) group, while those patients who recovered after a single treatment had a significantly higher abundance of the pathways in green. Fig. S4. Shown are the HUMAnN2 functional pathway abundances for all patients with both day 0 and day 7 samples. Fig. S5. The relative abundance of Akkermansia muciniphila ASV 2 is shown over time stratified by outcome. A) The patient IDs are marked in gray if symptoms resolved in a single dose of the study drug, in red if they required repeat intervention, and black if they come from the donor. B) Akkermansia muciniphila ASV2 abundance after re-intervention. Fig. S6. The PCA analysis from Main Fig. 2 is reproduced here via the dual principal component function of phyloseq, which uses Euclidean distances. The overall taxonomic composition is graphed in Panel A, while Panel B shows the directionality of the influence of individual taxa upon those samples. Fig. S7. A) The relative abundance of Clostridioides difficile is tracked here using species-specific toxin genes detected in metagenomic sequences via ShortBRED. The gene count was [file 13073_2021_843_MOESM1_ESM.pdf]

Fig. S1

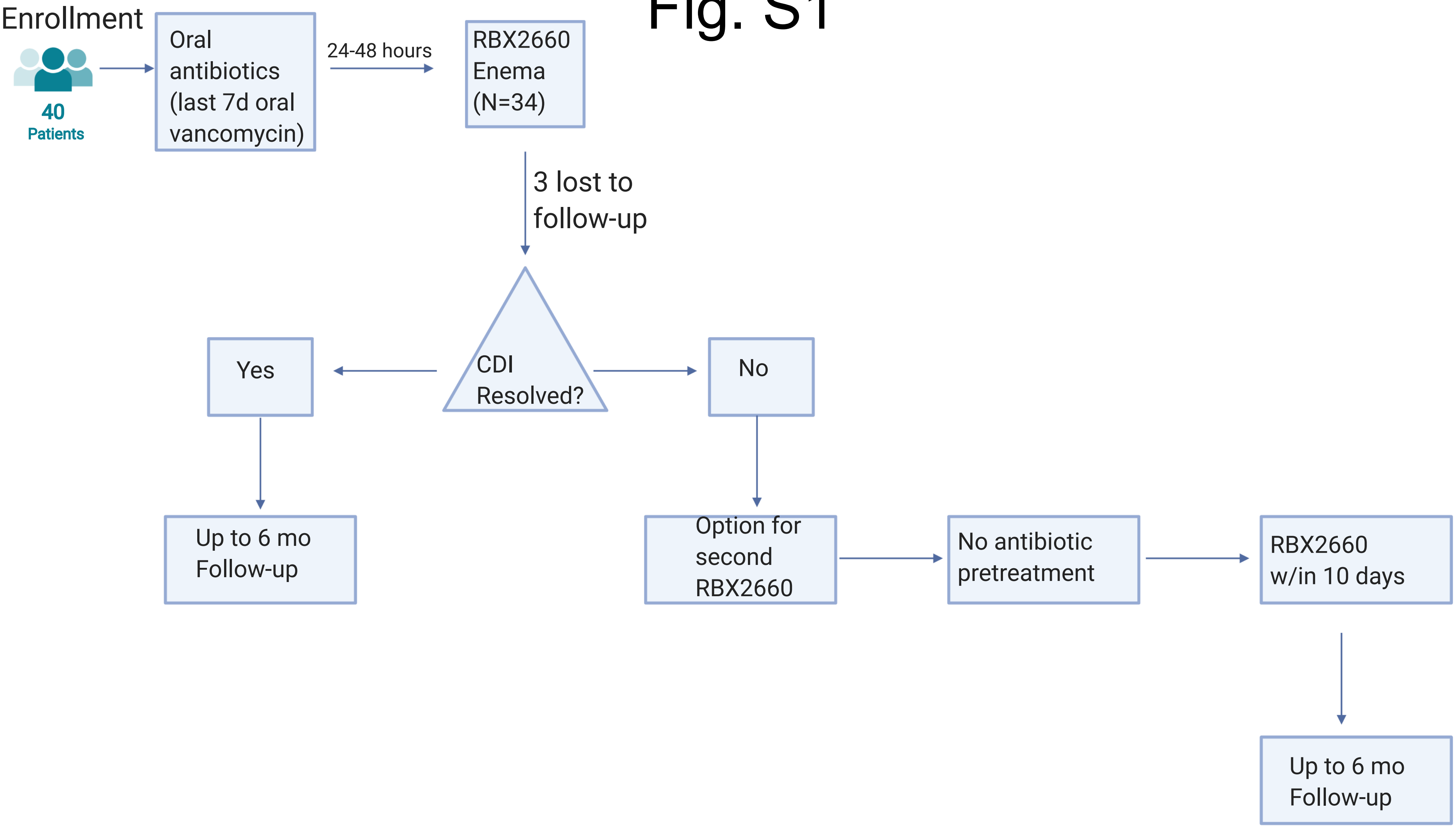

Enrollment
40
Patients
Oral antibiotics (last 7d oral vancomycin)
24-48 hours
RBX2660 Enema (N=34)
3 lost to follow-up
CDI Resolved?
Yes
No
Up to 6 mo Follow-up
Option for second RBX2660
No antibiotic pretreatment
RBX2660 w/in 10 days
Up to 6 mo Follow-up

Fig. S2

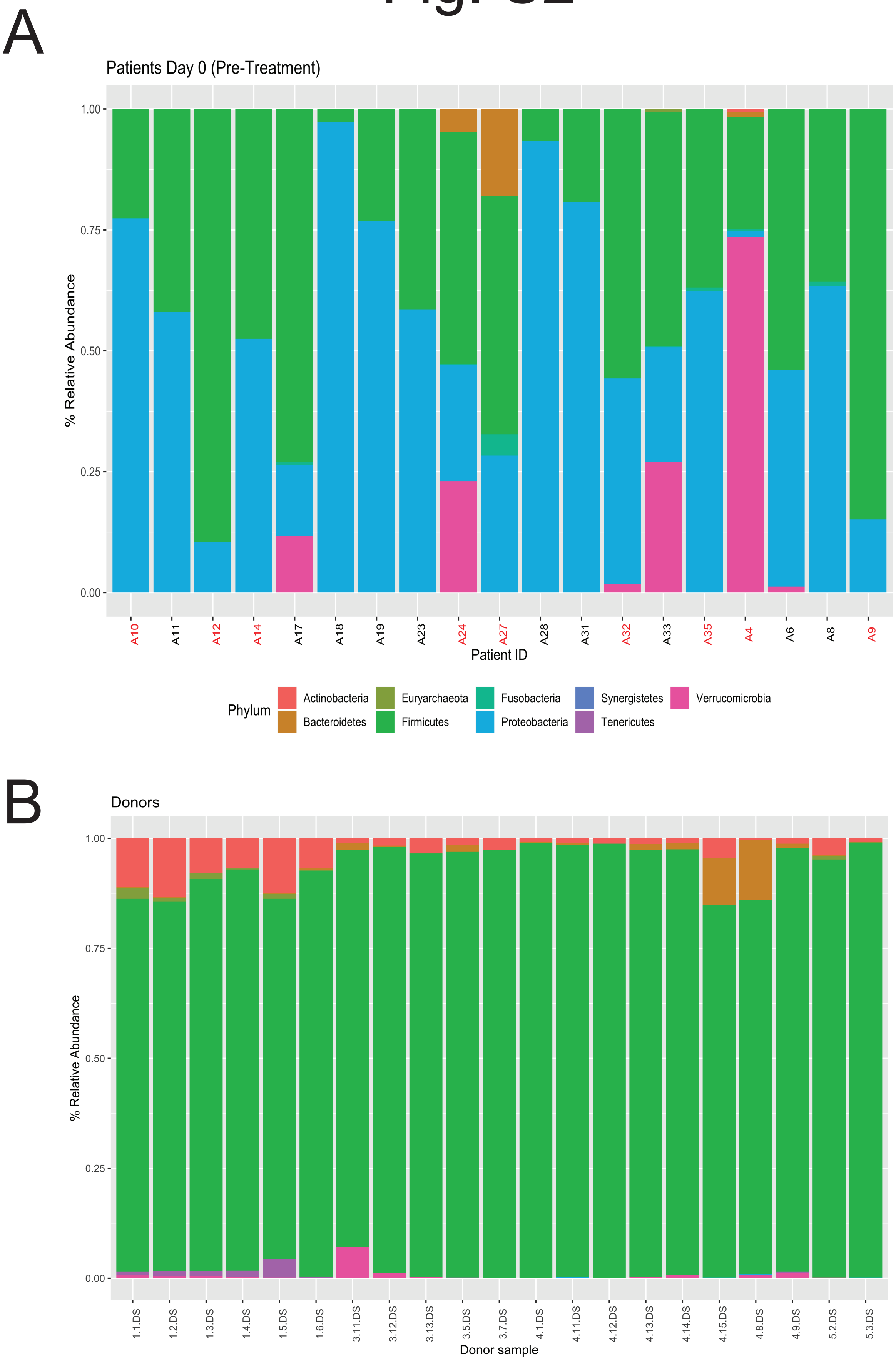
A
Patients Day 0 (Pre-Treatment)
% Relative Abundance
1.00
0.75
0.50
0.25
0.00
A10
A11
A12
A14
A17
A18
A19
A23
A24
A27
A28
A31
A32
A33
A35
A4
A6
A8
A9
Patient ID
Phylum
Actinobacteria
Bacteroidetes
Euryarchaeota
Firmicutes
Fusobacteria
Proteobacteria
Synergistetes
Tenericutes
Verrucomicrobia
B
Donors
% Relative Abundance
1.00
0.75
0.50
0.25
0.00
1.1.DS
1.2.DS
1.3.DS
1.4.DS
1.5.DS
1.6.DS
3.11.DS
3.12.DS
3.13.DS
3.5.DS
3.7.DS
4.1.DS
4.11.DS
4.12.DS
4.13.DS
4.14.DS
4.15.DS
4.8.DS
4.9.DS
5.2.DS
5.3.DS
Donor sample

Fig. S3

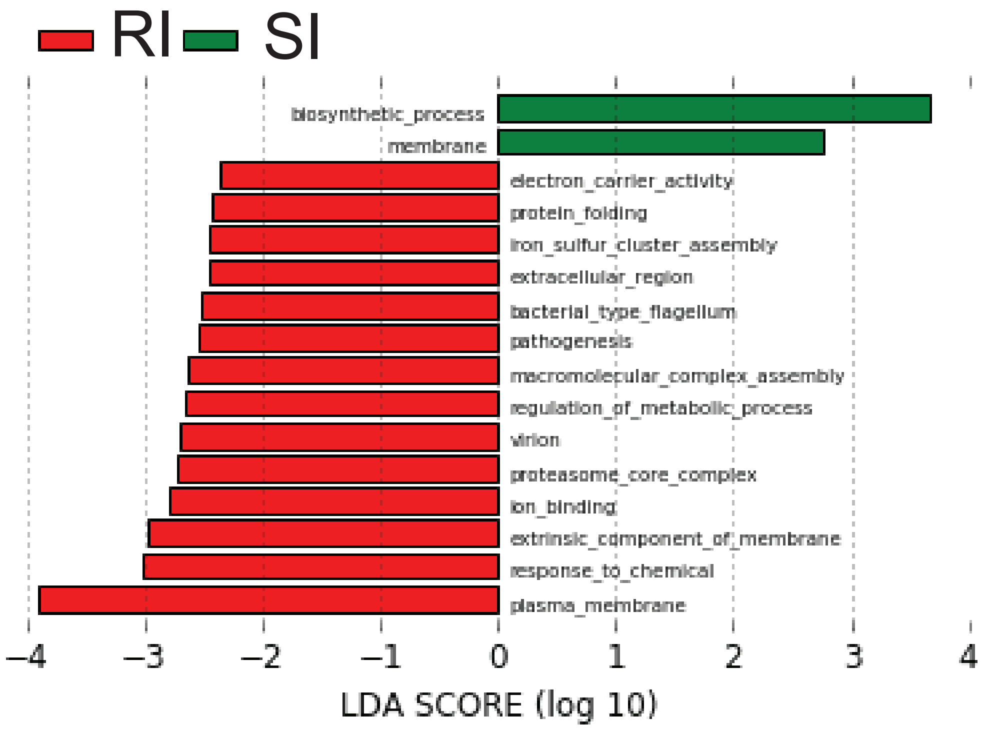

RI
SI
biosynthetic_process
membrane
electron_carrier_activity
protein_folding
iron_sulfur_cluster_assembly
extracellular_region
bacterial_type_flagellum
pathogenesis
macromolecular_complex_assembly
regulation_of_metabolic_process
virion
proteasome_core_complex
ion_binding
extrinsic_component_of_membrane
response_to_chemical
plasma_membrane
−4 −3 −2 −1 0 1 2 3 4
LDA SCORE (log 10)

Fig. S4

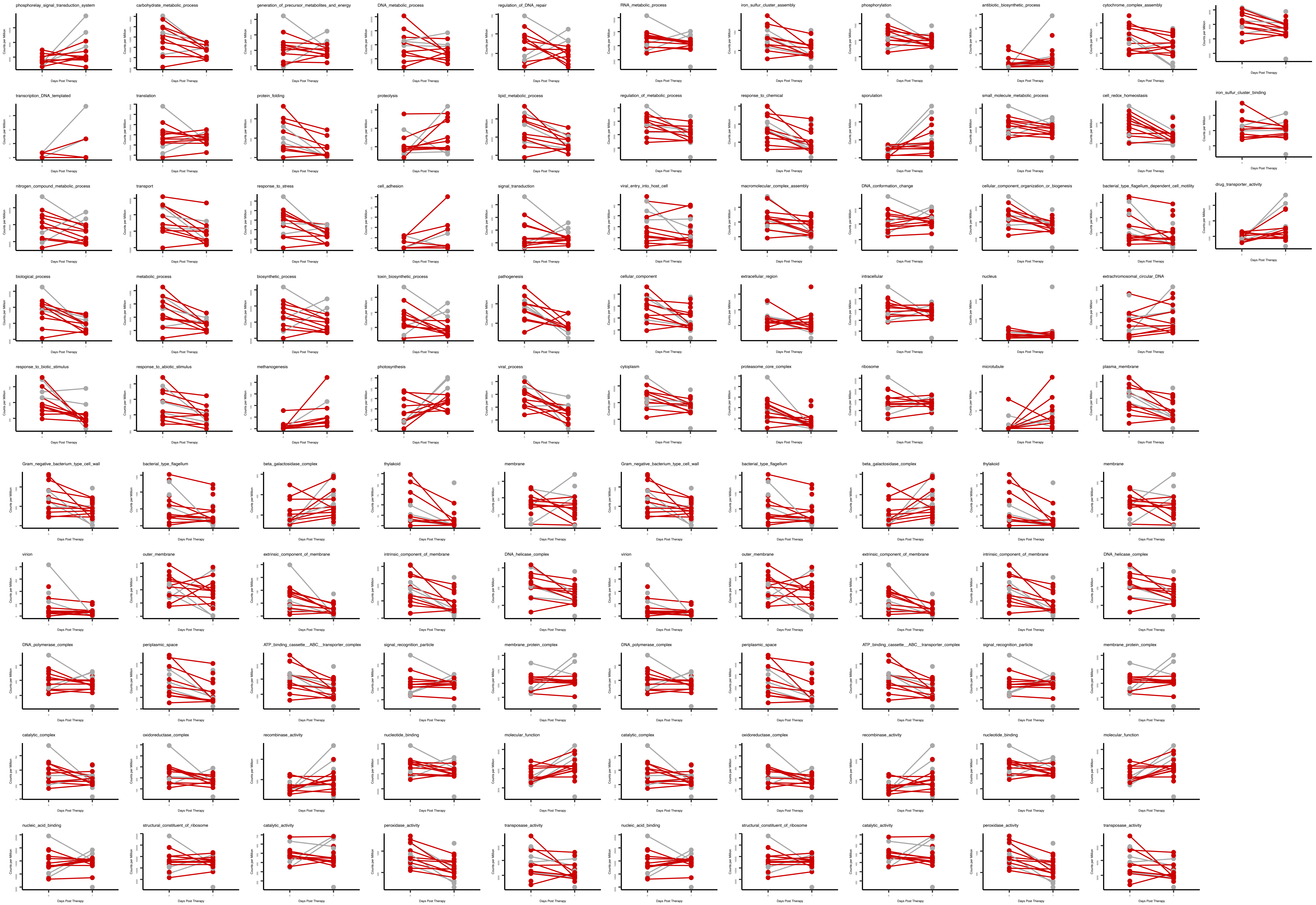
phosphorelay_signal_transduction_system
carbohydrate_metabolic_process
generation_of_precursor_metabolites_and_energy
DNA_metabolic_process
regulation_of_DNA_repair
RNA_metabolic_process
iron_sulfur_cluster_assembly
phosphorylation
antibiotic_biosynthetic_process
cytochrome_complex_assembly
coenzyme_binding
transcription_DNA_templated
translation
protein_folding
proteolysis
lipid_metabolic_process
regulation_of_metabolic_process
response_to_chemical
sporulation
small_molecule_metabolic_process
cell_redox_homeostasis
iron_sulfur_cluster_binding
nitrogen_compound_metabolic_process
transport
response_to_stress
cell_adhesion
signal_transduction
viral_entry_into_host_cell
macromolecular_complex_assembly
DNA_conformation_change
cellular_component_organization_or_biogenesis
bacterial_type_flagellum_dependent_cell_motility
drug_transporter_activity
biological_process
metabolic_process
biosynthetic_process
toxin_biosynthetic_process
pathogenesis
cellular_component
extracellular_region
intracellular
nucleus
extrachromosomal_circular_DNA
response_to_biotic_stimulus
response_to_abiotic_stimulus
methanogenesis
photosynthesis
viral_process
cytoplasm
proteasome_core_complex
ribosome
microtubule
plasma_membrane
Gram_negative_bacterium_type_cell_wall
bacterial_type_flagellum
beta_galactosidase_complex
thylakoid
membrane
virion
outer_membrane
extrinsic_component_of_membrane
intrinsic_component_of_membrane
DNA_helicase_complex
DNA_polymerase_complex
periplasmic_space
ATP_binding_cassette__ABC__transporter_complex
signal_recognition_particle
membrane_protein_complex
catalytic_complex
oxidoreductase_complex
recombinase_activity
nucleotide_binding
molecular_function
nucleic_acid_binding
structural_constituent_of_ribosome
catalytic_activity
peroxidase_activity
transposase_activity
Counts per Million
Days Post Therapy

Fig. S5

A

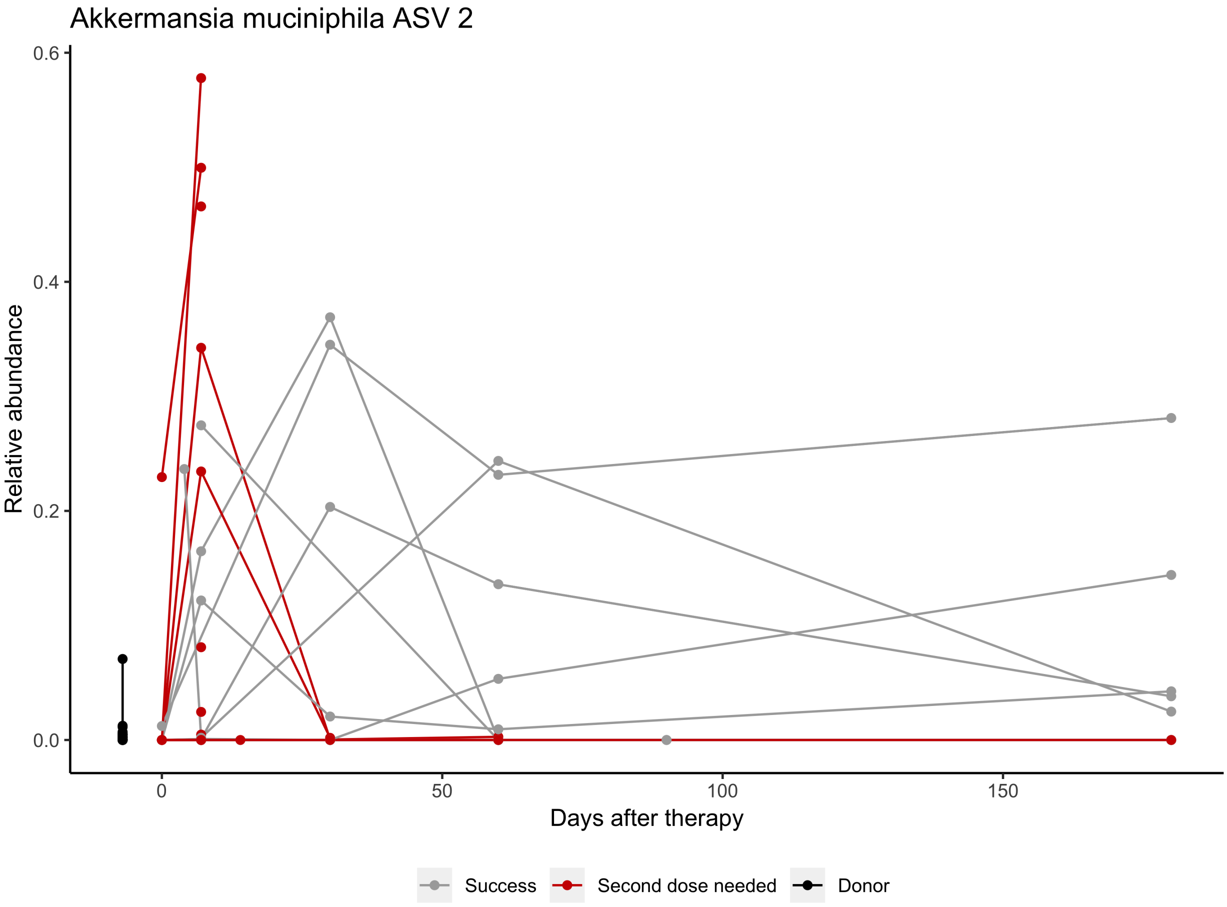

Akkermansia muciniphila ASV 2
Relative abundance
0.0
0.2
0.4
0.6
0
50
100
150
Days after therapy
Success
Second dose needed
Donor

B

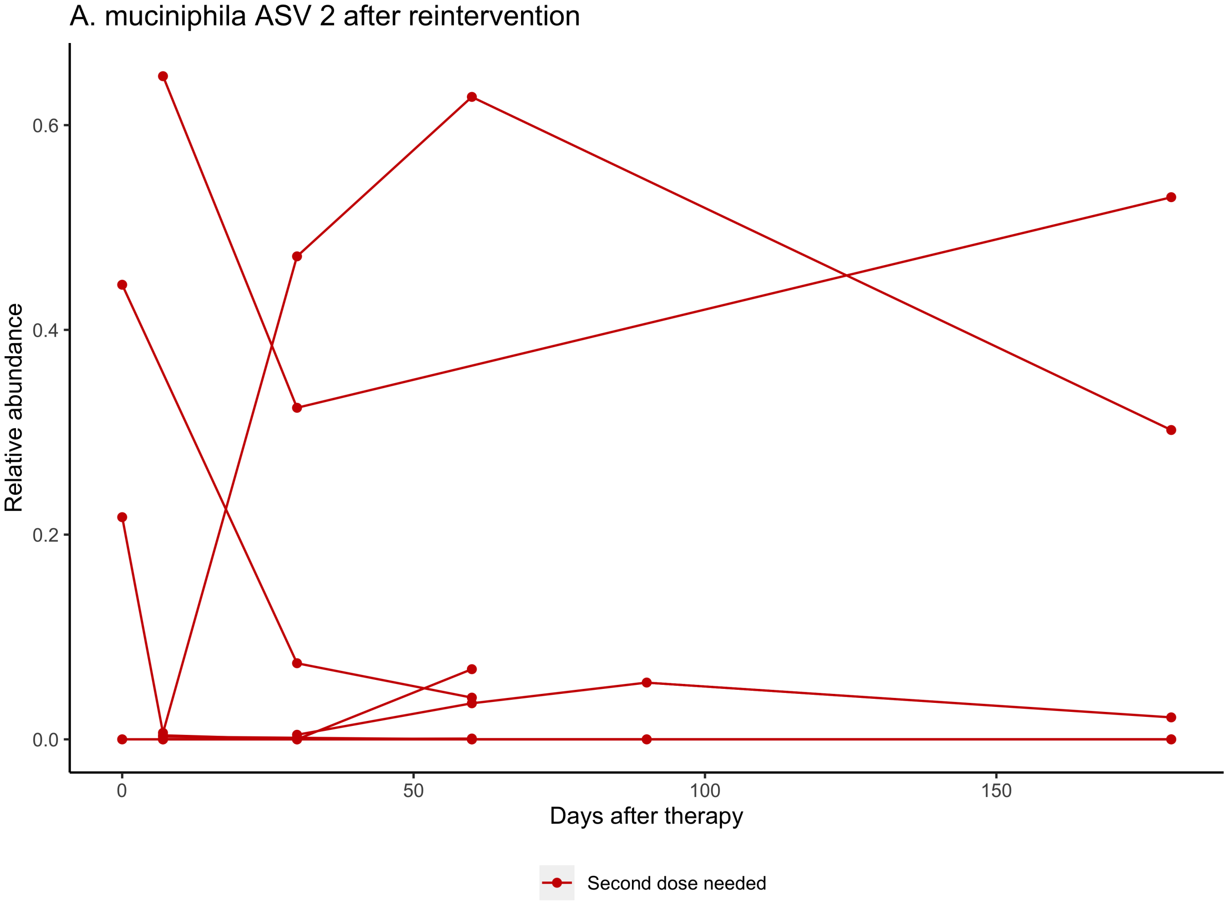

A. muciniphila ASV 2 after reintervention
Relative abundance
0.0
0.2
0.4
0.6
0
50
100
150
Days after therapy
Second dose needed

Fig. S6

A

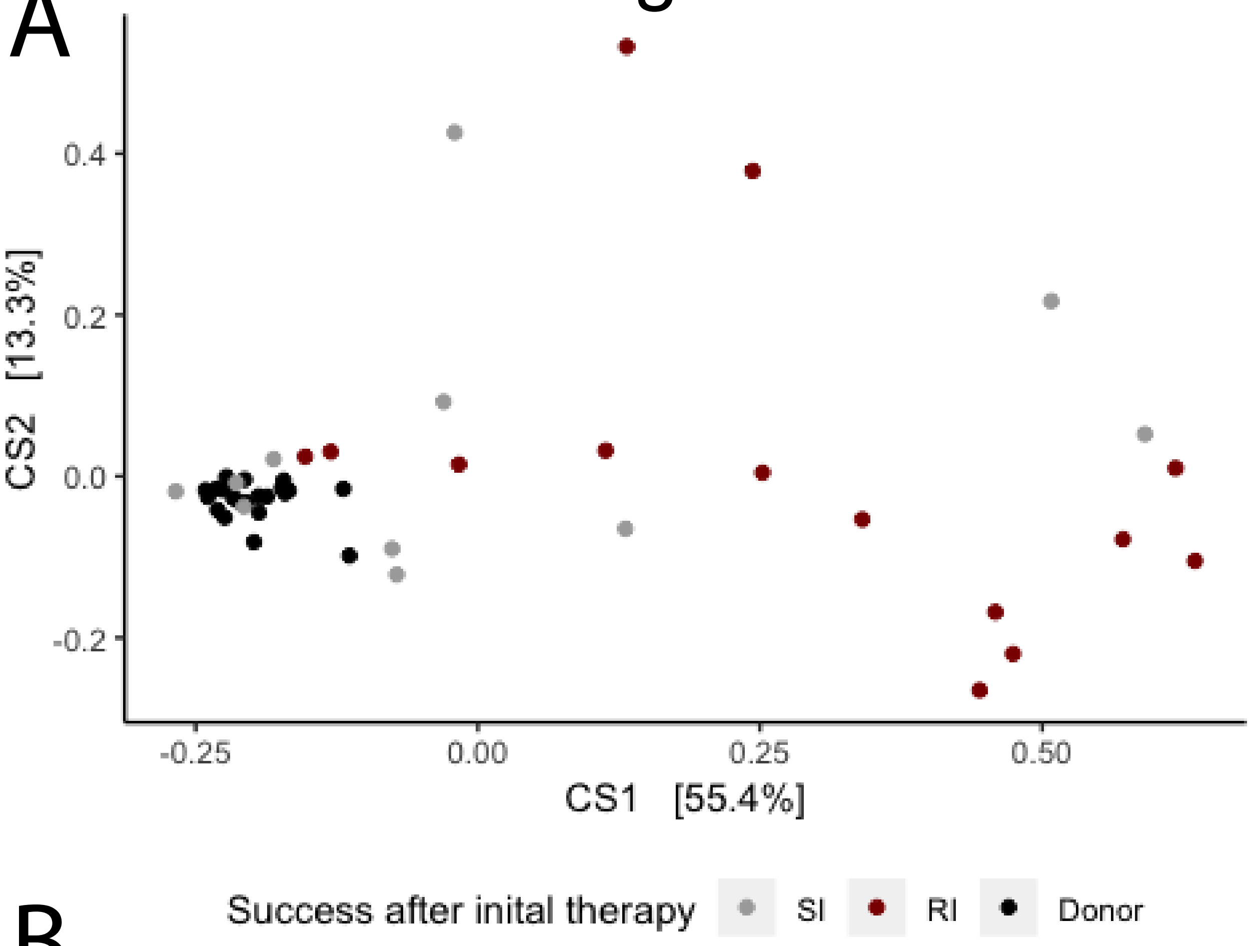
0.4
0.2
0.0
-0.2
CS2 [13.3%]
-0.25
0.00
0.25
0.50
CS1 [55.4%]
Success after inital therapy
SI
RI
Donor

B

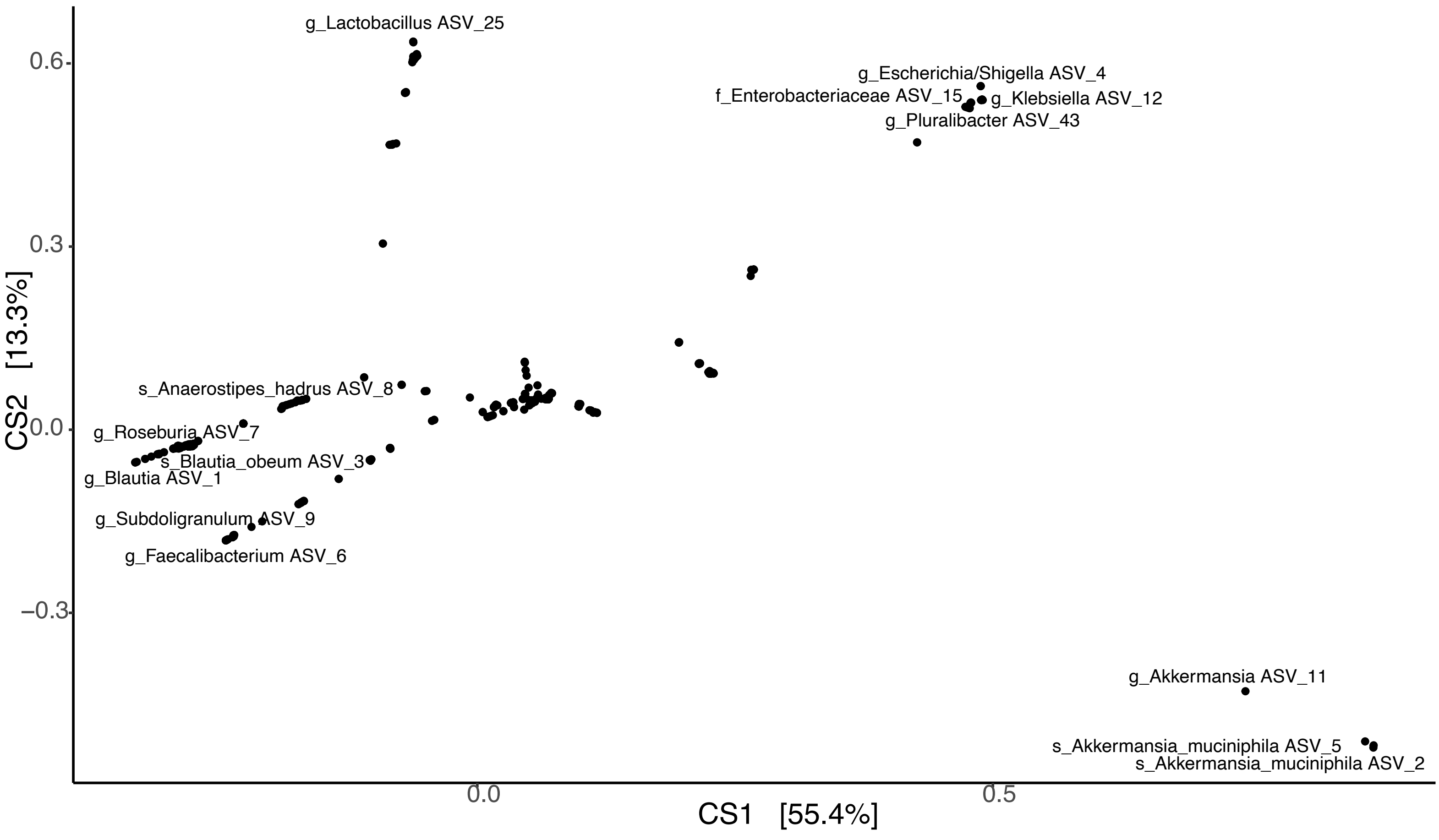
g_Lactobacillus ASV_25
g_Escherichia/Shigella ASV_4
f_Enterobacteriaceae ASV_15
g_Klebsiella ASV_12
g_Pluralibacter ASV_43
s_Anaerostipes_hadrus ASV_8
g_Roseburia ASV_7
s_Blautia_obeum ASV_3
g_Blautia ASV_1
g_Subdoligranulum ASV_9
g_Faecalibacterium ASV_6
g_Akkermansia ASV_11
s_Akkermansia_muciniphila ASV_5
s_Akkermansia_muciniphila ASV_2
0.6
0.3
0.0
−0.3
CS2 [13.3%]
0.0
0.5
CS1 [55.4%]

Fig. S7

A

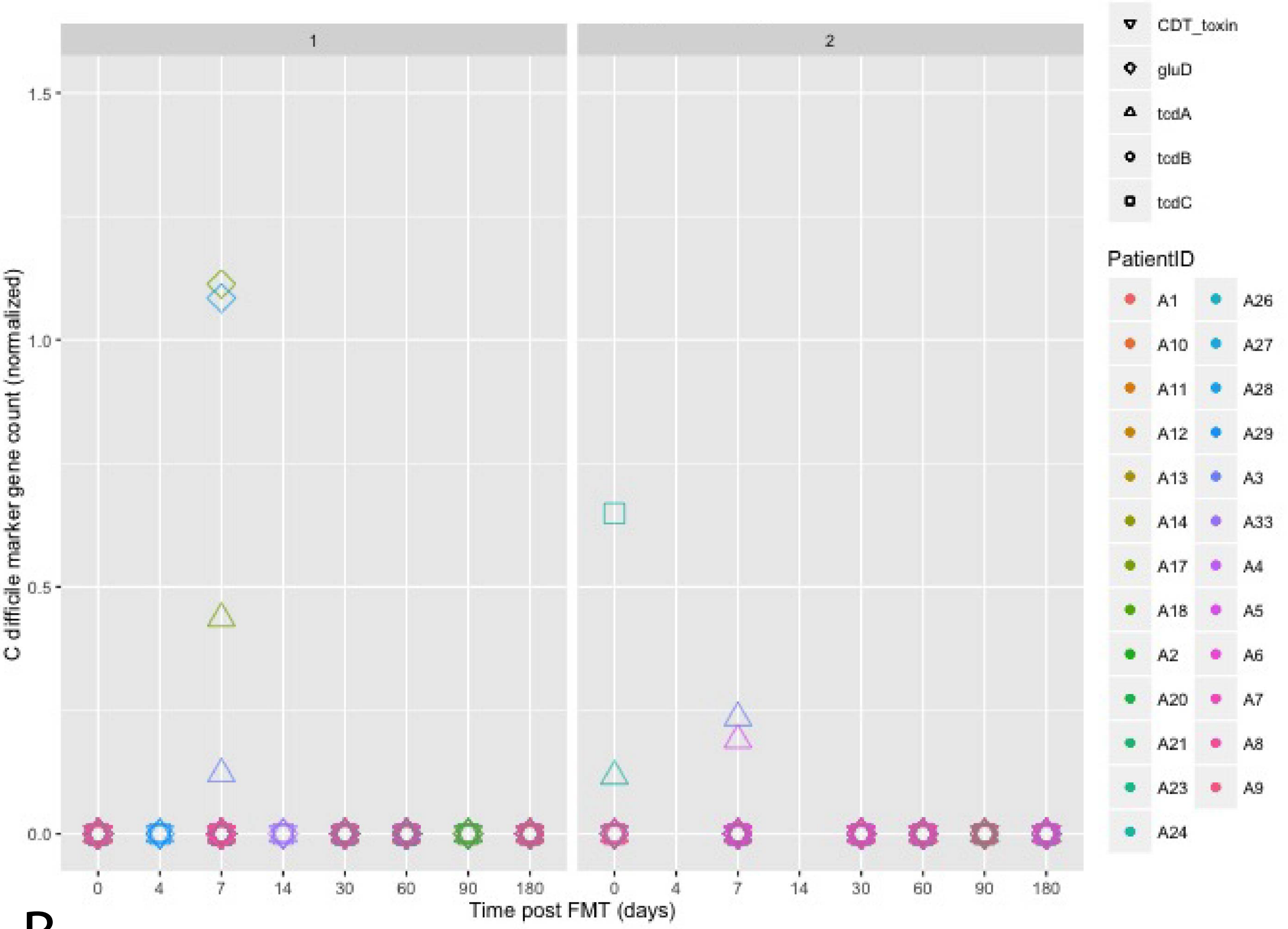
1
2
C difficile marker gene count (normalized)
1.5
1.0
0.5
0.0
0
4
7
14
30
60
90
180
Time post FMT (days)
CDT_toxin
gluD
tcdA
tcdB
tcdC
PatientID
A1
A10
A11
A12
A13
A14
A17
A18
A2
A20
A21
A23
A24
A26
A27
A28
A29
A3
A33
A4
A5
A6
A7
A8
A9

B

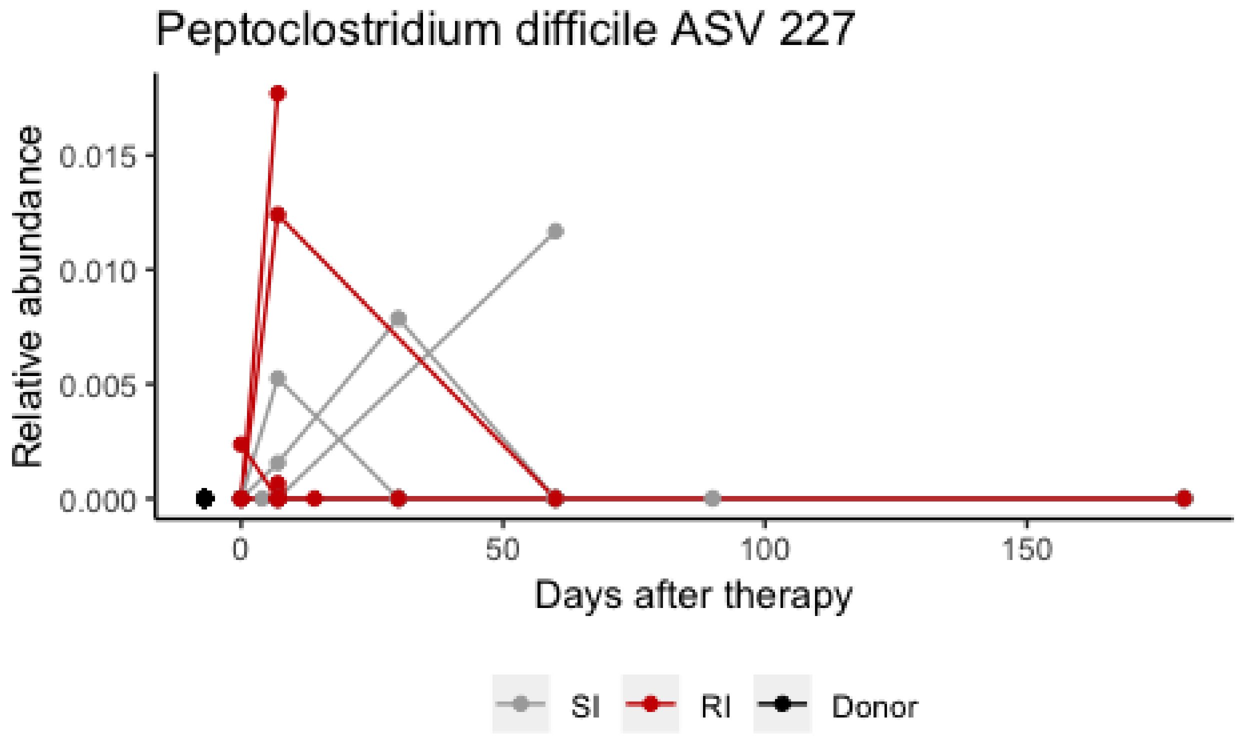
Peptoclostridium difficile ASV 227
Relative abundance
0.015
0.010
0.005
0.000
0
50
100
150
Days after therapy
SI
RI
Donor
