# Supplementary figures and images for: Microbiota restoration reduces antibiotic-resistant bacteria gut colonization in patients with recurrent Clostridioides difficile infection from the open-label PUNCH CD study
Source: Genome Med. 2021 Feb 16;13:28. doi: 10.1186/s13073-021-00843-9 (PMC7888090; doi:10.1186/s13073-021-00843-9)

# Fig. S8

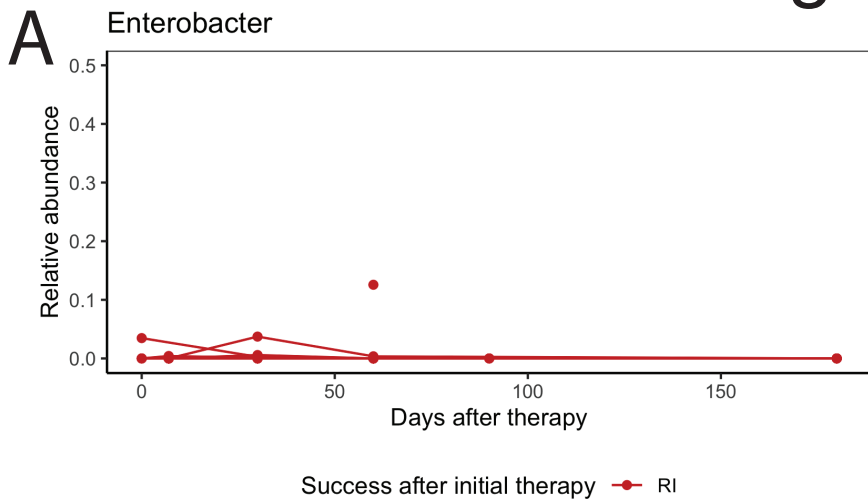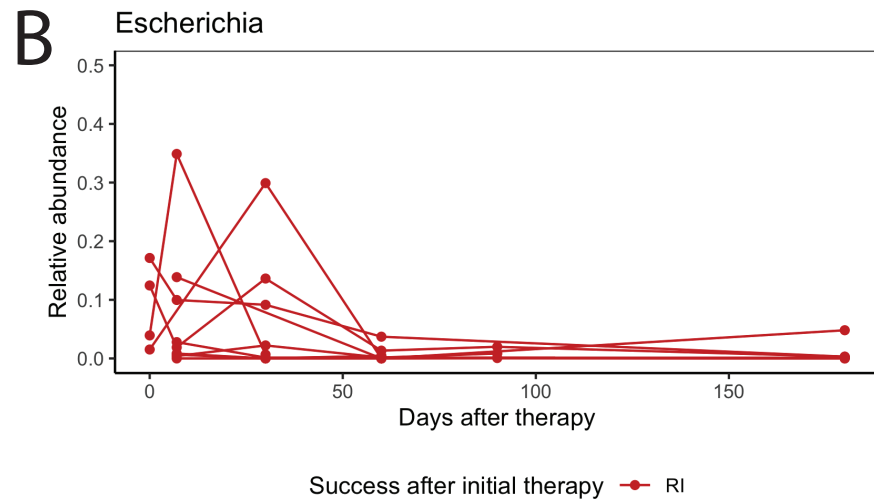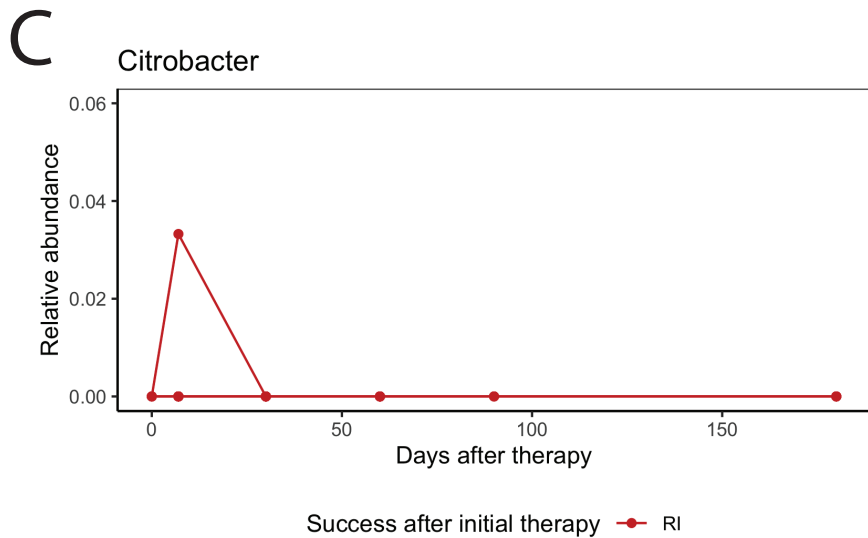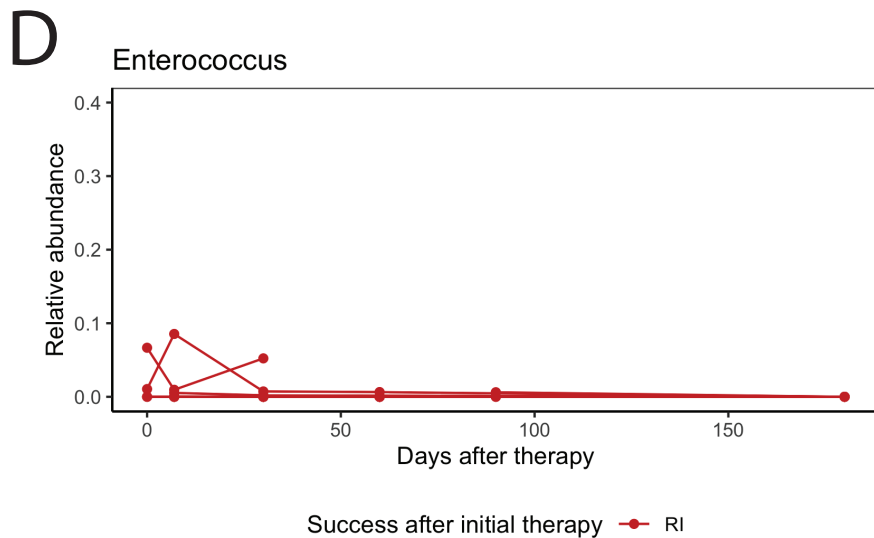

# Fig. S9

## A RI Group; FMT 1

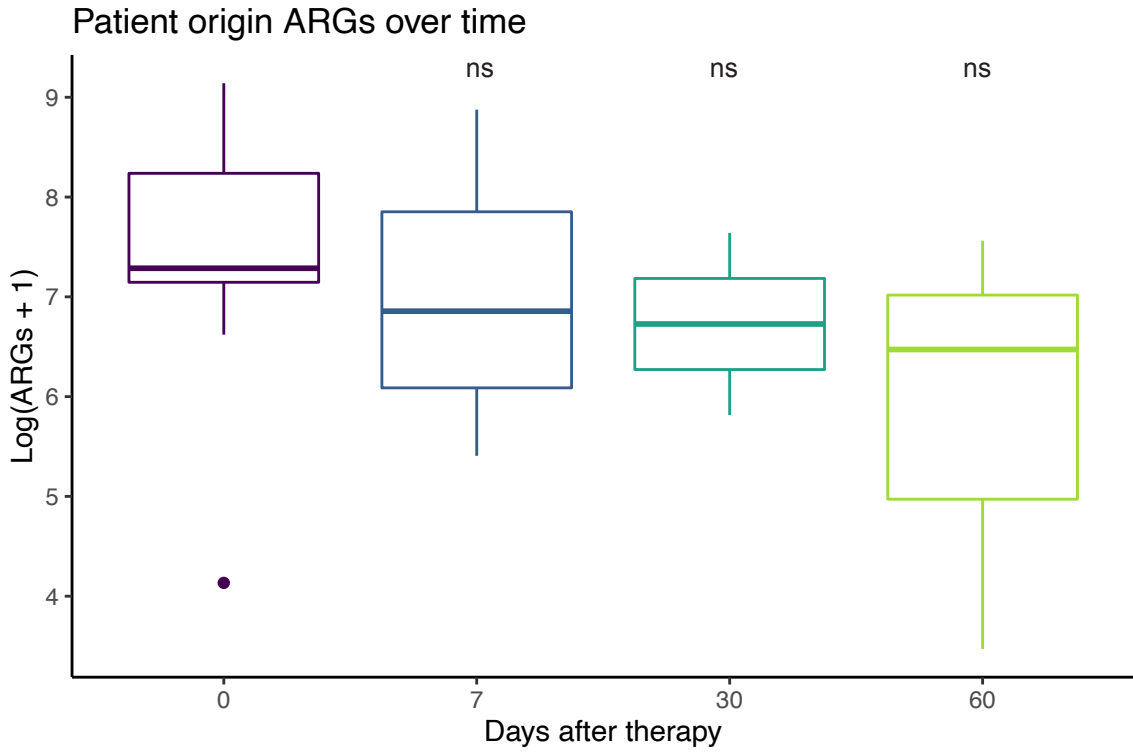

## B RI Group; FMT 2

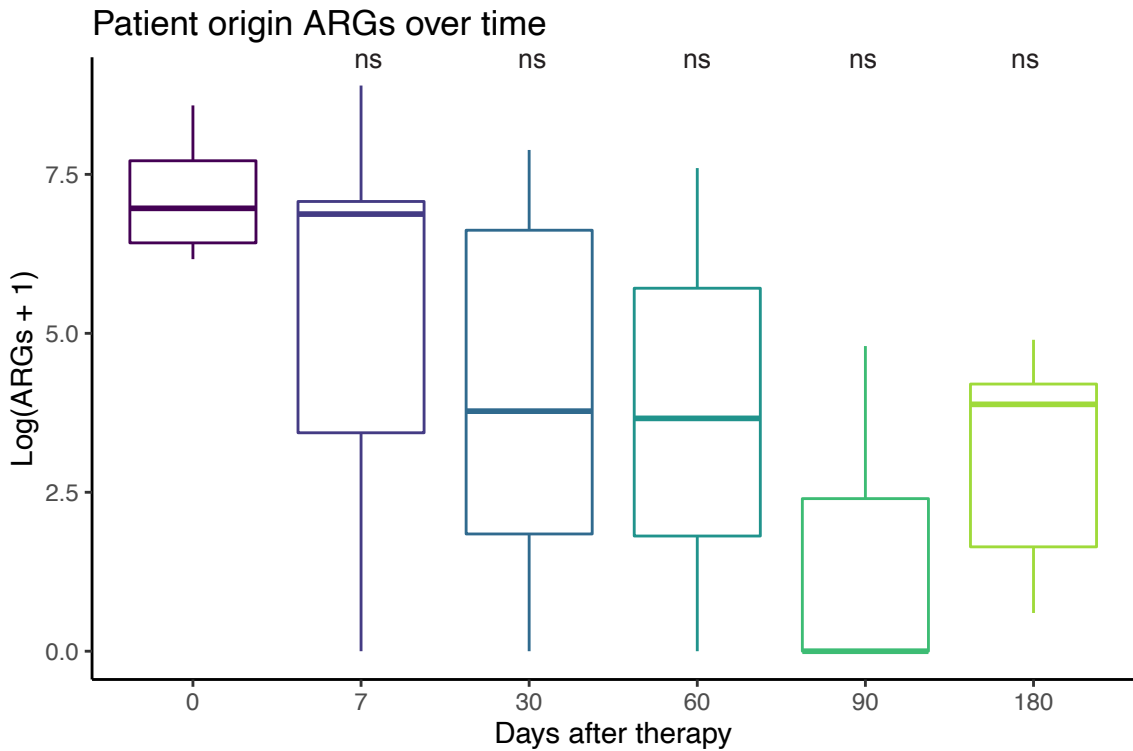

# Fig. S10

ARGs not present in patients Day 0 or in their donor

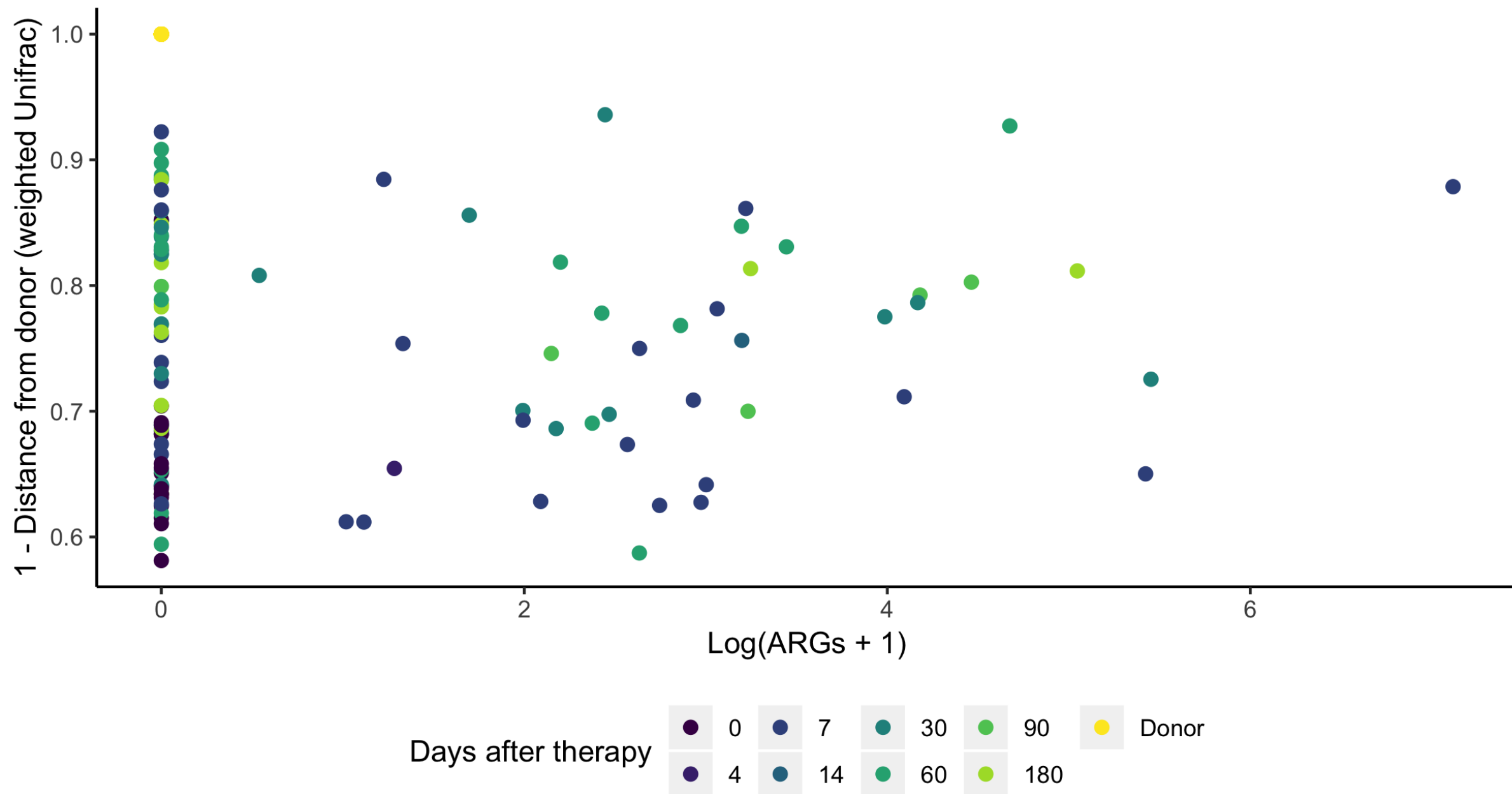

Supplement: Supplementary file 5 — Additional file 5 Fig. S8. Relative abundance of ASVs corresponding to A) Enterobacter, B) Escherichia, C) Citrobacter, and D) Enterococcus tracked temporally after second dose of RBX2660 in the RI group. Fig. S9. Patient origin ARGs over time after A) first RBX2660 and B) second RBX2660 in the RI group. All comparisons non-significant as determined by pairwise Wilcoxon with Benjamini Hochberg correction. n = 17 total patients with A) 27 and B) 45 samples. Fig. S10. Abundance of resistance genes in each metagenomic sample compared to their DFD for resistance genes that were not detected in patients’ day 0 samples or in the donors. For these genes, their abundance and the distance from donor are uncorrelated. [file 13073_2021_843_MOESM5_ESM.pdf]
